# Chemical constituents from a *Gynostemma laxum* and their antioxidant and neuroprotective activities

**DOI:** 10.1186/s13020-017-0136-y

**Published:** 2017-05-24

**Authors:** Ji Yeon Seo, Sang Kyum Kim, Phi Hung Nguyen, Ju Yong Lee, Pham Ha Thanh Tung, Sang Hyun Sung, Won Keun Oh

**Affiliations:** 1 Korea Bioactive Natural Material Bank, Research Institute of Pharmaceutical Sciences, College of Pharmacy, Seoul National University, Seoul, 08826 Republic of Korea; 2 College of Pharmacy, Chungnam National University, Daejeon, 34134 Republic of Korea; 3 College of Pharmacy, Chosun University, Gwangju, 61452 Republic of Korea

**Keywords:** *Gynostemma laxum*, Quercetin analogues, Antioxidant, Neuroprotection, Keap1

## Abstract

**Background:**

A few bioactivities of constituents from *Gynostemma laxum*, which has been collected in Vietnam, have been reported until now. There is no report about the effects of constituents from *G. laxum* although the nuclear factor (erythroid-derived 2)-like 2 (Nrf2)-mediated heme oxygenase-1 (HO-1) antioxidant defense system is involved in neuroprotection in the brain. Therefore, we investigated whether quercetin (**2**), benzoic acid (**10**) and their analogues (**1**, **3**–**9** and **11**) from *G. laxum* have the antioxidant and neuroprotective activities and also their underlying mechanism.

**Methods:**

To examine their neuroprotective and antioxidant activities, oxytosis, total oxidant scavenging capacity (TOSC), 2,7-dichlorofluorescein (DCFDA), dihydroethidium (DHE), antioxidant response element (ARE)-luciferase reporter gene assays, Western blot analysis, real time-PCR, immunocytochemistry and in silico 3D molecular docking simulation were performed.

**Results:**

The study of constituents using chromatographic techniques and spectroscopic analysis showed that *G. laxum* contained an abundance of quercetin (**2**), benzoic acid (**10**) and their analogues (**1**, **3**–**9** and **11**). Our data demonstrated that quercetin (**2**) and its analogue (**4**) among the constituents from *G. laxum* showed the strongest neuroprotective effect against oxytosis triggered by the excessive amount of glutamate. Compounds **2**, **4**, **6** and **11** exhibited reactive oxygen species (ROS) inhibitory and ARE transcriptional activities in immortalized hippocampal HT22 cell line. Among them, compound **4**, a second active compound, induced Nrf2/HO-1 activation. They were also fit stable onto the Tramtrack and Bric-à-Brac (BTB) domain of Kelch-like ECH-associated protein 1 (Keap1), a known Nrf2 inhibitor protein, based on the results of docking and interaction energies. Overall, these data suggest that –OH and –OCH_3_ groups of quercetin and its analogues are responsible for their neuroprotective effect.

**Conclusions:**

In summary, the major constituents of *G. laxum* had strong antioxidant and neuroprotective activities so that they could consider as a natural antioxidant supplement. Furthermore, *G. laxum* might be used beneficially in reducing oxidative complications with the further deep investigation in vivo.

**Electronic supplementary material:**

The online version of this article (doi:10.1186/s13020-017-0136-y) contains supplementary material, which is available to authorized users.

## Background

Oxidative stress can be defined as an imbalance between oxidants and the antioxidant system leading to oxidative damage at critical sites in tissues and cells [1]. The homeostasis between oxidants and antioxidants in the human body is maintained consistently under normal condition, and it is important for sustaining optimal physiology [2]. Recently, much interest has focused on the antioxidant activities of dietary components with the expectation that they may supplement the human natural defenses against various oxidant challenges [3].

Reactive oxygen species (ROS) such as the O_2_^−^, HO^−^ and other non-radicals oxygen analogues, e.g., H_2_O_2_ and ^1^O_2_ are generated by an integral part of metabolism [4]. Thus, antioxidants have been interested in biologists and clinicians as they may help to protect the human body against damage due to ROS [5, 6]. Antioxidants have also been used to preserve food quality mainly by prevention of oxidative deterioration of lipid constituent. When antioxidants added to lipids and lipid-containing foods, their shelf-life can increase retarding the process of lipid peroxidation. At present, the most commonly used antioxidants are butylated hydroxyanisole, butylated hydroxytoluene, propyl gallate, and *tert*-butylhydroquinone [7]. However, they have suspected to be responsible for their harmful effects on various organs such as brain [8] and liver [9]. Specifically, ROS has been regarded as one of the major reasons to occur the neurological diseases including Alzheimer’s [10], Parkinson’s [11] and Huntington’s diseases [12]. Therefore, development of more safer natural antioxidants from plant materials that can replace synthetic antioxidants has been of interest [13] as well as attenuation of ROS-mediated damages can be strategies to prevent or alleviate to neurodegenerative diseases [10, 14].

In the brain, nuclear factor (erythroid-derived 2)-like 2 (Nrf2)-mediated antioxidant enzymes including heme oxygenase-1 (HO-1) eliminate the intracellular ROS through the catabolism [15]. The recent many reports suggest that Kelch-like ECH-associated protein 1 (Keap1), which is an inhibitor protein of Nrf2, acts as a central regulator of the Nrf2-mediated cytoprotection and anti-inflammation. In this case, the N-terminal Broad complex, Tramtrack and Bric-à-Brac (BTB) domain of Keap1 plays important roles in sensing electrophilic antioxidants and interaction with the cullin 3 (Cul3)/E3 ubiquitin-protein ligase system which is an initial signal for proteasome degradation of Keap1 and Nrf2 complex. The C151 among the other cysteines in Keap1 has been considered as a responsible residue for interaction with electrophiles [16]. Thus, investigations of antagonists or interacting compounds into the active site of BTB domain in Keap1, particularly in C151, are also valuable approach for understanding the function of natural antioxidants.

The genus *Gynostemma*, which belongs to the Cucurbitaceae family, is comprised of about 21 species including *G. pentaphyllum, G. laxum,* and *G. pubescens*. These plants distribute over large areas in Japan, China, and also Vietnam [17]. Of the genus *Gynostemma*, many studies have been focused on the *G*. *pentaphyllum* with dammarane-type saponins as the discovery of new activities for treatment of metabolic disease [18–20], anti-cancer activity through the inhibition of proliferation [17], and learning and memory disorder [21]. However, the scientific study of chemical constituents and biological activities against neuroprotection with the *G. laxum* has not reported so far.

During the purpose of screening for antioxidants from natural plants, we found that an EtOAc fraction partitioned from 70% ethanolic extract of *G. laxum* exhibited significant antioxidant activities. Thus, we report for the first time on the characterization of the chemical constituents of *G. laxum* and their neuroprotective and antioxidant properties in vitro radical scavenging systems and neuronal cell models.

## Methods

### Plant material

The dried aerial parts of *G. laxum* were collected at Bac Kan province, Northern Vietnam in spring, 2013. A voucher specimen was deposited in the Medicinal Herbarium of Hanoi University of Pharmacy (HUP) with the accession number HNIP: 18500/16. Plant material was identified as *G. laxum* based on morphological characteristics. For DNA authentification of *G. Laxum*, total DNA was extracted from 200 mg of fresh plant leave using a Dneasy Plant Mini Kit (QIAGEN, Germany) with some modifications. PCR amplification and sequencing were conducted by Macrogen Inc. (Seoul, Korea) using a pair of primers: ITS1-F (5′-TCCGTAGGTGAACCTGCGG-3′), ITS4-R (5′-TCCTCCGCTTATTGATATGC-3′). The sequence of ITS region of sample and reference sequence of *G. laxum* (KF269126) from Genebank NCBI was aligned by Geneious Alignment (65% similarity). The combined length of the entire ITS region (ITS1, 5.8S and ITS2) from taxa was analyzed with a 535 nucleotides. As ITS region of *G. laxum* are highly matched with the sequence over 99.4% from Genebank (KF269126), the plant material was finally identified as *G. laxum* based on morphological characteristics and DNA barcoding methods (Additional file 2).

### Total oxidant scavenging capacity (TOSC) assay

A slight modification of the method developed by Regoli and Winston was used to determine the TOSC of the quercetin and its analogues [22]. Peroxy radicals were produced by thermal homolysis of 60 mM 2,2′-azobis-amidinopropane (ABAP) in 200 mM potassium phosphate buffer, pH 7.4, at 35 °C. Reactions with 0.4 mM α-keto-γ-methiobutyric acid (KMBA) were carried out in 10 mL rubber septa-sealed vials in a final reaction volume of 1 mL. Ethylene production was measured by gas chromatographic analysis of 200 μL aliquots taken from the headspace of vials at indicated intervals during the reaction. Total ethylene formation was quantified from the area under the kinetic curve. Samples were monitored in sequence at 15 min intervals over a time course of 60 min. Analyses were performed with a Shimadzu-2010 (Shimadzu Corp., Tokyo, Japan) gas chromatograph equipped with a SPB-1 capillary column (30 m × 0.32 mm × 0.25 μm) and a flame ionizing detector (FID). The oven, injection, and FID temperatures were 60, 180, and 180 °C, respectively. Helium, at a flow rate of 30 mL/min, was used as the carrier gas.

### Quantification of TOSC

The TOSC value for each concentration of the sample was calculated as following:

1 $$ {\text{TOSC}} = 100  -  \left( {{{\smallint {\text{SA}} } / {\smallint {\text{CA}} }} \times 100} \right). $$

Here, ∫SA and ∫CA are the integrated areas from the sample reaction and control reaction, respectively. Thus, a sample with no oxy-radical scavenging capacity would give an area equal to the control (∫SA/∫CA = 1) and a resulting TOSC value of 0. However, as the ∫SA approaches 0, the hypothetical TOSC value approaches 100. sTOSC values were obtained from the slope of the linear regression lines for the TOSC curves and rTOSC values were quantified by dividing the sTOSC value of the sample by that obtained of trolox, as shown below:

2 $$ {\text{rTOSC}} = {{{\text{sTOSC}}\left( {\text{sample}} \right)}/ {{\text{sTOSC}}\left( {\text{trolox}} \right)}} $$

### Molecular docking simulation

Possible docking modes between the ligands and active site 1 of BTB domain of Keap1 (PDB code: 4CXT) or its mutant at C151W were monitored by CDOCKER protocol in CHARMm force fields. CDOCKER energy indicates the ligand–protein docking energy. Ligand interacting affinity was expressed as CDOCKER interaction energy and interacting bonds such as hydrogen bond, pi–alkyl, pi–sulfur and Van der Waals using Discovery Studio 4.0 (Accelrys, San Diego, CA).

### 2,7-Dichlorofluorescein (DCFDA) and dihydroethidium (DHE) assays

Oxidative stress was quantified in cells by DCFDA [23] or DHE [24] (Invitrogen Thermo Fisher Scientific, Rockford, IL) assays according to the previously reported methods with slight modifications.

### Antioxidant response element (ARE)-reporter gene assay

HT22-ARE or SH-SY5Y-ARE cells [25] were plated onto 12-well plates at a density of 3 × 10^5^ cells/well for 6 h, and then the cells were incubated in DMEM containing 1% FBS for another 6 h. After sample treatment, the cells were incubated for 16 h, and the collected cell lysates were used for measuring the luciferase activity according to the protocol guided by the manufacturer (Promega Corp., WI).

### Western blot analysis

Thirty μg of proteins were loaded onto the SDS-PAGE gel, the gel was run at 200 V for 1–2 h, and then the proteins were transferred to Immobilion^®^-P 0.45 μm PVDF transfer membrane (Millipore, Billerica, MA). After blocking the non-specific proteins on the membrane, the primary antibodies for Nrf2 (H-300, Santa Cruz), HO-1 (sc-1797, Santa Cruz), Lamin B (sc-474, Santa Cruz) and β-actin (ab6276, Abcam) were loaded and were incubated for more than 18 h at 4 °C. After washing the membranes, secondary antibodies such as horseradish peroxidase-conjugated anti-rabbit, anti-goat or anti-mouse-immunoglobulin G were loaded and were incubated for 2 h at room temperature. The protein bands were visualized through the reaction with chemiluminescent substrates from Thermo Scientific (Rockford, IL) using Image Quant™ LAS4000 imaging system from GE Healthcare Life Sciences (Little Chalfont, UK). The relative protein expressions were calculated by using ImageJ software (National Institutes of Health, Bethesda, MD).

### Immunocytochemistry

HT22 cells were seeded onto a 35 mm cell culture plates and the cells were treated with DMEM with 1% FBS, PS and 20 μM of compound 4 for 12 h. After fixation and permeabilization steps, the cells were incubated with 1% BSA solution. The cells were reacted with primary antibodies (1:200 ratio, 4 °C), and were sequentially incubated with secondary antibodies conjugated with FITC or Texas red (1:500 ratio) for 2 h. The nucleus was stained with 1 μg/mL DAPI for 1 min. After washing the cells, the cells were mounted by using the Vectashield mounting medium (Vector Laboratories, Burlingame, CA). The confocal immunofluorescence images were taken by Confocal microscopy TCS SP8 (Leica, Wetzlar, Germany).

### Real-time PCR

The mRNA extraction was performed by the Trizol method and then the mRNA was immediately synthesized to cDNA using Maxim RT PreMix (random primer) manufactured from iNtRON Biotechnology (Seongnam, Korea). The cDNA was subjected to do the real time-PCR. Primers were used to amplify HO-1 (forward: CAGCCCCACCAAGTT and reverse: GGCGGTCTTAGCCTCTTCTGT) and 18S (forward: GCTTAATTTGACTCAACACGGGA and reverse: AGCTATCAATCTGTCAATCCTGTC). The real time-PCR was done using StepOne Real-Time PCR System provided by Thermo Fisher Scientific (Waltham, MA) at this time condition: 10 min incubation at 95 °C and 40 cycles of 15 s at 95 °C and 1 min at 60 °C. 2X SYBR green and ROX dye (Bioneer, Daejeon, Korea) were used to measure the relative mRNA expression level.

### Statistical analysis

The significance of results was analyzed by the one-way analysis of variance (ANOVA) using Tukey’s post hoc or the Newman–Keuls multiple range tests using SPSS statistics 23 (SPSS, Inc., Chicago, IL, USA).

### Information of experimental design and resources

Details of our experimental design, statistics, and all resources used in this study were included in the Minimum Standards of Reporting Checklist (Additional file 1).

## Results

### Isolation and structure determination of chemical constituents from *G. laxum*

The EtOAc fr. of *G. laxum* was subjected to a succession of chromatographic procedures including silica gel, Shephadex LH-20, RP-C18, and HPLC to afford 11 compounds (**1**–**11**). Chemical structures of isolated compounds were identified by ^1^H, ^13^C, and HMBC NMR analyses, and comparing their physicochemical and spectroscopic data with those published in the literature (Additional file 2). Compound **2** was obtained as a yellow powder and gave a positive ferric chloride reaction. The UV displayed two maximum bands at 260 and 381 nm, characteristic of a flavon-3-ol. In addition, the ^1^H and ^13^C NMR spectra showed a typical ABX aromatic spin system for ring B [*δ*_H_ 7.81 (1H, d, 2.0, H-2′), 6.98 (1H, dd, 2.0, 8.5, H-5′), and 7.68 (1H, d, 8.5, H-6′)], with corresponding carbons at *δ*_C_ 120.2, 117.3, and 112.1, respectively. Two oxygenated quaternary carbons at *δ*_C_ 148.5 (C-3′) and 147.7 (C-4′) supported this observation. Therefore, compound **2** was determined to be quercetin [26]. In comparison, the ^1^H and ^13^C NMR spectroscopic patterns of compounds **2**, **3**, and **4** were identical except only for the signals assignable for a methoxy group in **3** and **4** [*δ*_H_ 3.93 (3H, s), *δ*_C_ 56.4–56.5]. The methoxy group was found to be attached at C-3′ in compound **3** and at C-4′ in compound **4** by an HMBC experimental analyses. Thus, compounds **3** and **4** were characterized as quercetin-3′-methyl ether and quercetin-4′-methyl ether, respectively. As compounds **5** and **6** possessed two methoxy moieties in the structure, one methoxy group was attached to C-3, and the other one was substituted at C-3′ in **5** and C-4′ in **6**, respectively. Ermanin (**7**) and kaempferol-3-methyl ether (**9**) were also similar with **5** and **6** bearing a methoxy group at C-3 (*δ*_C_ 131.3–132.6). The significant differences in the ^1^H NMR spectra of **7** and **9**, as compared to **5** and **6**, were the pattern of substitution in the B-ring at *δ*_H_ 8.05–8.25 (2H, br, d, 9.0) and 6.90–7.12 (2H, br, d, 9.0), indicative of H-2′/6′ and H-3′/5, respectively. Therefore, compound **9** was characterized as kaempferol-3-methyl ether, and compound **7** as to be ermanin, respectively.

**Fig. 1:**
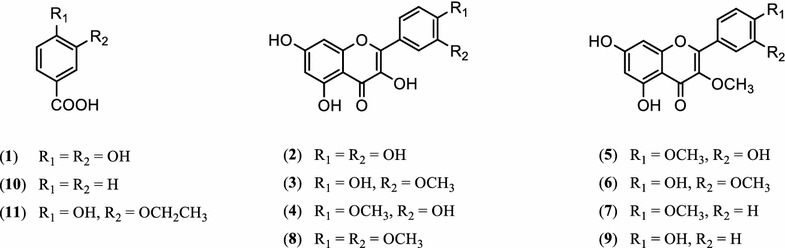
Chemical structures of isolated compounds

Phytochemical study suggests that the aerial part of *G. laxum* is an abundant source of natural phenolics, which were identified as quercetin (**2**), quercetin analogues (**3**–**9**), benzoic acid (**10**) and benzoic acid analogues (**1**, **11**). It is well known that phenolics have a wide impact on the living system and that the most interested property of phenolics is antioxidant property [27]. Isolated compounds were determined as 3,4-dihydroxybenzoic acid (**1**), quercetin (**2**), quercetin-3′-methyl ether (**3**), quercetin-4′-methyl ether (**4**), quercetin-3,4′-dimethyl ether (**5**), quercetin-3,3′-dimethyl ether (**6**), ermanin (**7**), quercetin-3′,4′-dimethyl ether (**8**), kaempferol-3-methyl ether (**9**), benzoic acid (**10**), and 3-ethoxy-4-hydroxybenzoic acid (**11)** (Fig. 1).

Quercetin (**2**): Yellow powder; m.p. (uncorrected) 312–318 °C, FeCl_3_ test: positive; UV (MeOH) λ_max_ nm: 260, 381; ^13^C NMR (125 MHz, acetone-*d*_6_): δ_C_ 178.2, 165.3, 164.1, 158.9, 153.3, 148.5, 147.7, 132.8, 123.9, 120.2, 117.3, 112.1, 104.6, 95.8, 94.9; ^1^H NMR.

Quercetin-4′-methyl ether (**4**): Yellowish powder; FeCl_3_ test: positive; ^13^C NMR (125 MHz, acetone-*d*_6_): δ_C_ 178.1, 164.5, 163.1, 158.8, 154.5, 147.4, 147.0, 132.3, 124.1, 120.7, 117.8, 112.9, 105.6, 96.7, 95.6, 56.4.

### Oxytosis inhibitory activities of isolated compounds from *G. laxum*

**Fig. 2:**
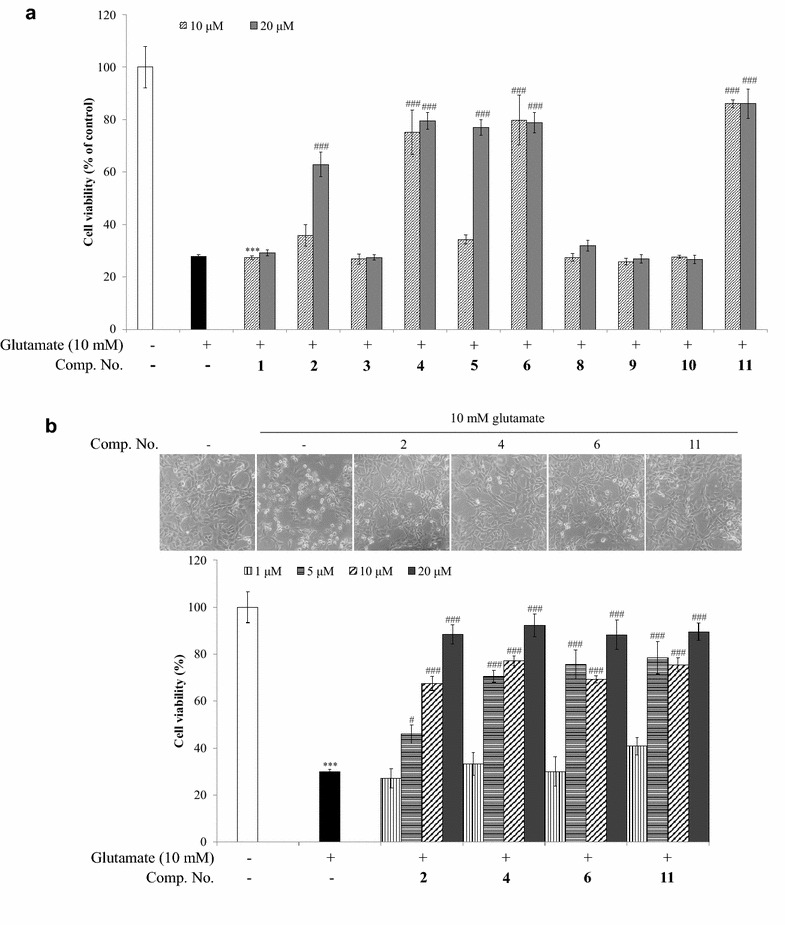
Neuroprotective effects of isolated compounds from *G. laxum* in HT22 cells. **a** Cells were treated with each isolated compound from *G. laxum* at 10 or 20 μM in the presence or absence of 10 mM glutamate for 12 h. Values expressed to AV ± SD. N = 3, the significance was presented as ***p < 0.001 compared to the control, ^###^ *p* < 0.001 compared to the glutamate-treated cell group. **b** Cells were treated with compounds **2**, **4**, **6**, and **11** at 1, 5, 10, and 20 μM in the presence of 10 mM glutamate for 12 h. Values expressed to AV ± SD. N = 3, the significance was presented as ***p < 0.001 compared to the control, ^#^ *p* < 0.05 and ^###^ *p* < 0.001 compared to the glutamate-treated cell group

The neuroprotective effects of the isolated compounds **1**–**11** were evaluated by an oxytosis assay which is a type of cytotoxicity assays with the extremely high concentration of glutamate treatment on the HT22 cells. Quercetin (**2**), quercetin analogues (**4**–**6**) and compound **11** at 20 μM exerted significant cytoprotective effect against cell death induced by 10 mM glutamate. Among them, compounds **2**, **4**, **6** and **11** had shown the protective effects at treatment concentrations at a range of 1, 5, 10, and 20 μM. The cells did not get damage at the treatment of compounds **2**, **4**, **6** and **11** whereas condensed and shrunk cells were observed at a single glutamate treatment (Fig. 2).

### ROS inhibitory activities of isolated compounds from *G. laxum*

**Fig. 3:**
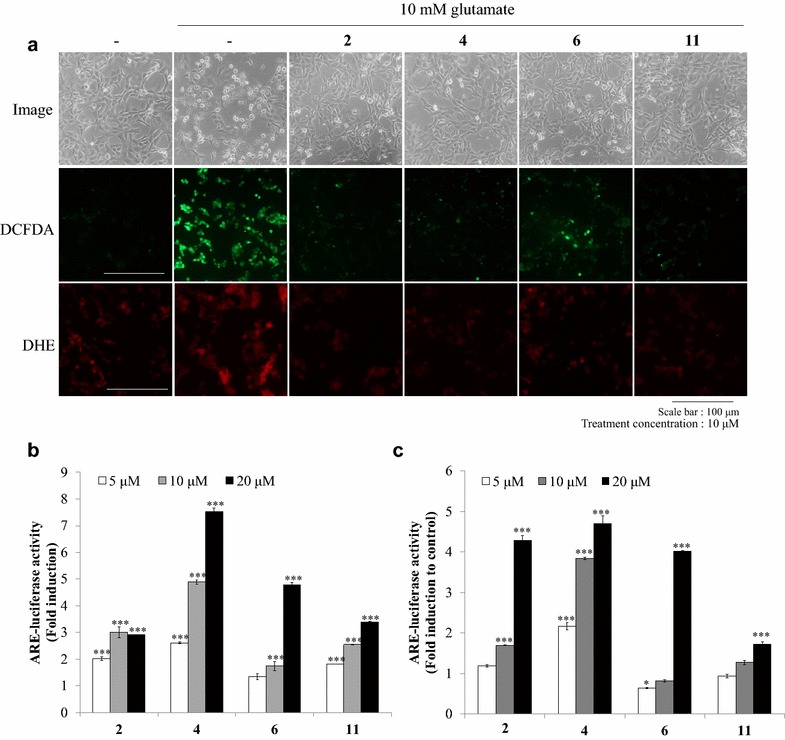
Effects of compounds **2**, **4**, **6**, and **11** on ROS inhibitory and ARE transcription inducing activities. **a** DCFDA and DHE assays were visualized as the bright field cell images, intracellular ROS and superoxide anion levels by compounds **2**, **4**, **6**, and **11** at 10 μM for 10 h plus 10 mM glutamate. The magnification is ×200. **b** ARE-luciferase reporter gene assay was performed using HT22-ARE cells. Cells were treated with compounds **2**, **4**, **6**, and **11** at 5, 10, and 20 μM for 16 h. The specific methods are described in “Methods” section. Values expressed to AV ± SD. N = 3, the significance was presented as *p < 0.05 and ***p < 0.001 compared to the control

To understand responsible mechanism for the neuroprotective effect of compounds **2**, **4**, **6** and **11**, intracellular ROS and superoxide anion were measured by staining with molecular probes such as DCFDA for total ROS or DHE for superoxide anion. Intracellular ROS and superoxide anion produced by glutamate were effectively eliminated by compounds **2**, **4** and **11**. Interestingly the ROS-removing effect of compounds **2** and **4** was higher than compound **6** (Fig. 3a).

### ARE transcriptional activities of isolated compounds from *G. laxum*

To elucidate whether quercetin (**2**) or its analogues (**4** or **6**) attenuate an intracellular ROS production and whether these effects are involved in ARE transcriptional activity, ARE reporter gene assay was performed using HT22-ARE and SH-SY5Y-ARE cells which contain ARE-luciferase reporter gene. The results demonstrated that compounds **2**–**4**, **6** and **11** induced ARE transcriptional activity over 2.5 times of relative value compared to the control at 20 μM in HT22-ARE and SH-SY5Y cells (Fig. 3b, c; Additional file 2).

### Nrf2-mediated HO-1 induction by compound 4

**Fig. 4:**
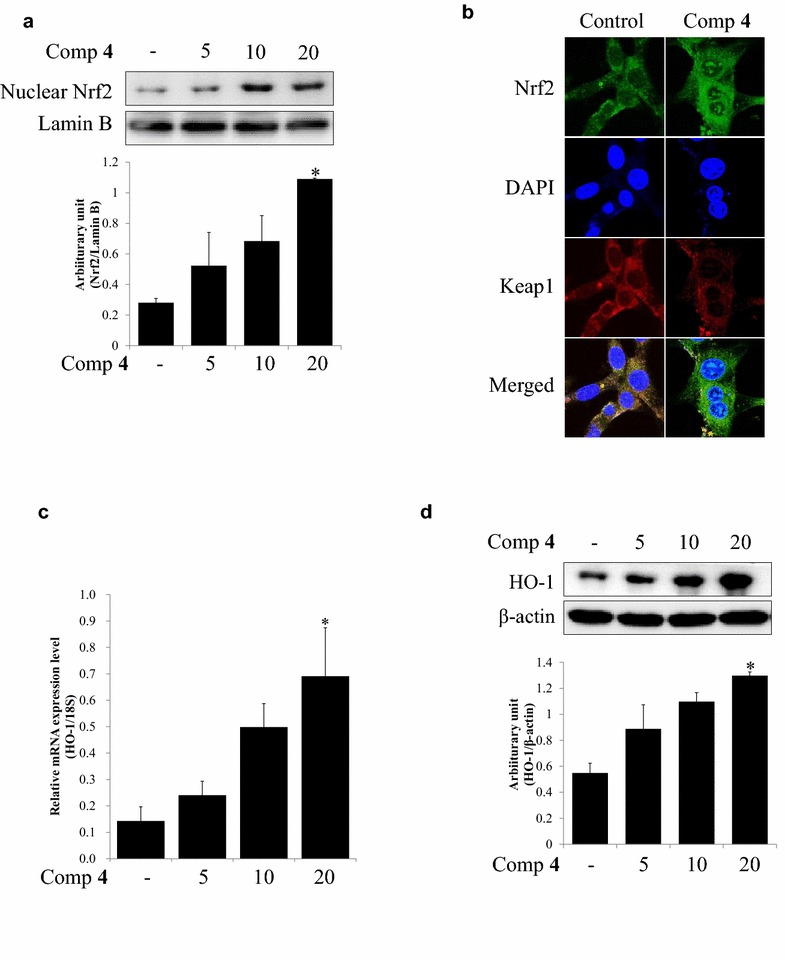
Nrf2-mediated HO-1 induction by compound **4** in HT22 cells. **a** Nuclear Nrf2 accumulation by compound **4** at 5, 10, and 20 μM was analyzed by Western blotting. **b** Translocation of Nrf2 into nucleus by compound **4** at 20 μM was determined by the immunocytochemistry procedure using confocal fluorescence microscope. **c** Real-time PCR evaluated the mRNA expression of HO-1 by treatment of compound **4** at 5, 10, and 20 μM for 24 h. Values expressed to AV ± SD. N = 3, the significance was presented as *p < 0.05 compared to the control. **d** HO-1 expression pattern by treatment of compound **4** at 5, 10, and 20 μM for 24 h. Values expressed to AV ± SD. N = 2, the significance was presented as *p < 0.05 compared to the control

The Nrf2/HO-1 related study about compound **4** (quercetin-4′-methyl ether), which was shown the excellent neuroprotective and ARE gene transcriptional activities, has not been elucidated so far. As shown in Fig. 4, compound **4** enhanced the Nrf2 accumulation in nucleus and translocation of Nrf2 from cytoplasm to nucleus at 20 μM. It also increased mRNA and protein expressions of HO-1 in hippocampal neuroblastoma HT22 cells by concentrations in a range of 5–20 μM (Fig. 4). These data indicate that compound **4** have HO-1 inducing effects mediated by Nrf2 activation.

### TOSC activities of quercetin and its analogues **(2**–**9**) from *G. laxum*

In the present study, when quercetin (**2**) and quercetin analogues (**3**–**9**) reduced ethylene production in the presence of each sample in a concentration-dependent manner. The slopes of the regression lines were calculated from the linear portion of TOSC vs. concentration of the quercetin and quercetin analogues used, and the sTOSC and rTOSC values against peroxy radicals are listed in Table 1. Ethylene generation from KMBA oxidation upon thermal homolysis of ABAP was markedly inhibited by the quercetin and quercetin analogues. Quercetin (**2**) showed the greatest sTOSC value against peroxy radicals followed in decreasing order by compounds **4**, **3**, **8**, **6**, **9**, **5**, and **7**. Quercetin with two hydroxy groups in B ring displayed the highest sTOSC value of 11.76 ± 1.27 TOSC/μM, and compound **4** with a methoxy moiety substituted at C-4′ of the B ring (5.54 ± 0.40 TOSC/μM) showed significantly greater antioxidant capacities against peroxy radicals than Trolox, a positive antioxidant (2.88 ± 0.14 TOSC/μM), respectively. Compound **3** with a methoxy group at C-3′ and **8** with two methoxy groups in B ring (3.39 and 3.32 TOSC/μM, respectively) showed slightly lower activity than compounds **2** and **4**. Both methoxy substitutions at the 3-hydroxy group in C ring and 3′-hydroxy group of catechol in B ring (compound **5**) were significantly decreased the sTOSC values. In addition, compound **6** showed more strong scavenging capacity than compound **5** (significantly different by Student’s *t* test, *p* < 0.05), with the same skeleton compound **9** also showed stronger effect than **7** (Table 1). Thus, the methoxy group may play a role in decreasing activity when attached to C-4′ position of the catechol.

**Table 1:** TOSC values of quercetin and quercetin analogues against peroxyl radicals

Compound	Concentration (μM)	TOSC value	sTOSC value (TOSC/μM)	rTOSC value
**2**	0.625	3.2	11.76 ± 1.27^a^	4.08
	1.25	11.5	(R^2^ = 0.9772)	
	2.5	28.4		
	5	41.7		
	10	71.8		
	20	94.4		
**3**	2.5	7.7	3.39 ± 0.34^c^	1.18
	5	27.3	(R^2^ = 0.9717)	
	10	38.1		
	20	68.6		
	40	92.0		
**4**	1.25	8.0	5.54 ± 0.40^b^	1.92
	2.5	12.1	(R^2^ = 0.9894)	
	5	28.2		
	10	72.0		
	20	93.6		
	40	97.4		
**5**	10	−0.8	0.44 ± 0.05^d^	0.15
	20	7.1	(R^2^ = 0.9781)	
	40	12.9		
	80	35.4		
**6**	5	4.2	1.04 ± 0.03^d^	0.36
	10	11.5	(R^2^ = 0.9977)	
	20	21.2		
	40	41.2		
	80	69.8		
**7**	10	0.5	0.12 ± 0.02^d^	0.04
	20	−0.1	(R^2^ = 0.9268)	
	40	2.8		
	80	9.0		
**8**	1.25	0.4	3.22 ± 0.15^c^	1.12
	2.5	8.3	(R^2^ = 0.9957)	
	5	14.5		
	10	32.5		
	20	54.5		
	40	88.6		
	80	95.8		
**9**	5	4.4	0.53 ± 0.07^d^	0.18
	10	6.65	(R^2^ = 0.9686)	
	20	11		
	40	13.2		
	80	27.2		

The TOSC values of each concentration are mean from three independent measurements. The sTOSC values were obtained from the slope of the linear regression line for the TOSC curves, and the rTOSC values were determined by dividing the sTOSC value of the sample by that of Trolox. The specific TOSC values of Trolox against peroxyl radicals were 2.88 ± 0.14 TOSC/μM. Values with different letters are significantly different from each other (ANOVA followed by Newman–Keuls multiple range test, *p* < 0.05)

### Molecular docking simulation of isolated compounds to BTB domain of Keap1

**Fig. 5:**
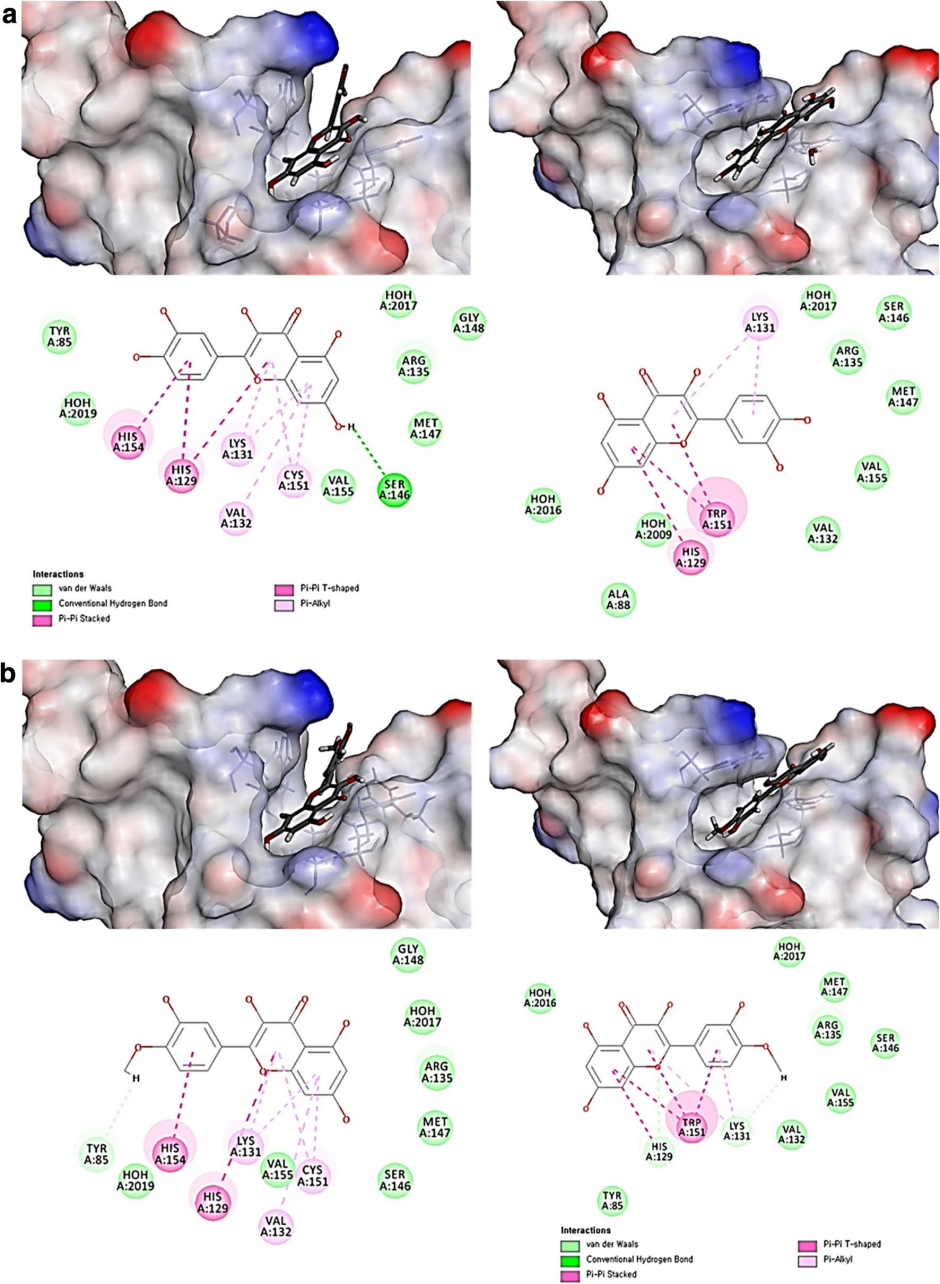
Molecular docking and interaction images of compounds **2** and **4** with Keap1. **a** Quercetin docked in BTB domain of Keap1 (*left-up side*) and mutant BTB domain at C151W of Keap1 (*right-up side*). The interpolated structures were shown in this figure. The 2D diagram of ligand interactions was illustrated at *down side*. **b** Quercetin-4′-methyl ether docked in BTB domain of Keap1 (*left-up side*) and mutant BTB domain at C151W of Keap1 (*right-up side*). The interpolated structures were shown in this figure. The 2D diagram of ligand interactions was illustrated at *down side*

We investigated whether isolated compounds from *G. laxum* can be docked in BTB domain of Keap1 and also can interact with amino acids positioned at BTB domain including the C151 residue by comparing with the docking and docking interaction energies of sulforaphane (SFN) as the positive control (Table 2). As results, quercetin had the lowest CDOCKER and CDOCKER interaction energies which indicate the binding affinity to Keap1. Consequently, the compounds fit stably onto the BTB domain and it was ordered by compounds **2**, **3**, **4**, and **8** at CDOCKER and **5**, **6**, **9**, and **7** at CDOCKER interaction energies respectively. The compounds **2**, **3**, and **4** docked to the BTB domain better than SFN and the quercetin (**2**) and its analogues (**3**–**9**) interacted with the BTB domain better than SFN. The C151 interacted with rings A and C of compounds **2** and **4**, the most active compounds, by pi–alkyl bonds in the contrast of other quercetin analogues. To investigate the involvement of C151 in docking of quercetin and its analogues to BTB domain of Keap1, C151 was mutated to W151. As results, CDOCKER and CDOCKER interaction energies of quercetin and its analogues were decreased by C151W mutation whereas benzoic acid and its analogues did not lower the CDOCKER and CDOCKER interaction energies. Interestingly, C151W mutation changed the space of active site of BTB domain in Keap1 so that the binding heads of compounds **2** and **4** against binding site also changed by opposite site. The changes in binding style lead to the interaction characteristics of compounds **2** and **4** in BTB domain of Keap1. In particular, the results illustrated the blockage of pi–pi stacked or pi–alkyl interactions between active compounds (**2**, **4**) and H154 or V132 (Table 2; Fig. 5). In summary, when C151 was mutated to W151 in Keap1, compounds **2**–**9** had shown higher energy differences than those of SFN at both CDOCKER and CDOCKER interaction energies. These results indicate that the influence of C151 residue of Keap1 keeps on the interaction between compounds and Keap1 protein.

**Table 2:** Molecular docking and interaction energies of isolated compounds from *G. laxum*

	CDOCKER energy		CDOCKER interaction energy	
	Wild type	C151W mutation	Wild type	C151W mutation
SFN	−20.7 ± 0.5^c^	−19.1 ± 1.0^e,^*	−19.1 ± 0.4^bc^	−17.8 ± 0.9^a^
1	−19.3 ± 0.7^c^	−22.5 ± 0.4^g,^**	−18.1 ± 0.6^b^	−21.1 ± 0.4^b,##^
2	−26.6 ± 0.2^e^	−21.4 ± 0.4^fg,^**	−28.9 ± 0.1^de^	−23.8 ± 0.4^c,##^
3	−23.8 ± 0.5^d^	−18.2 ± 0.7^de,^**	−29.2 ± 1.2^de^	−24.1 ± 0.8^c,#^
4	−24.7 ± 0.4^de^	−20.8 ± 0.1^f,^**	−30.8 ± 0.5^e^	−26.5 ± 0.3^e,#^
5	−16.2 ± 0.4^b^	−11.0 ± 1.1^b,^**	−30.1 ± 0.6^de^	−26.1 ± 1.1^de,#^
6	−15.3 ± 0.4^ab^	−9.3 ± 0.6^a,^***	−29.4 ± 0.5^de^	−24.0 ± 1.0^c,##^
7	−14.0 ± 0.3^a^	−9.0 ± 0.2^a,^***	−29.7 ± 0.9^de^	−24.5 ± 0.1^cd,#^
8	−19.9 ± 0.1^c^	−14.1 ± 0.2^c,^***	−31.3 ± 0.4^e^	−25.0 ± 0.3^cde,###^
9	−14.8 ± 0.5^ab^	−9.8 ± 0.4^ab,^**	−27.7 ± 0.4^d^	−23.3 ± 1.0^c,#^
10	−13.6 ± 1.0^a^	−17.4 ± 0.2^d,^*	−14.7 ± 1.1^a^	−18.6 ± 0.1^a,#^
11	−21.0 ± 1.7^c^	−22.4 ± 0.4^g^	−21.4 ± 1.8^c^	−23.2 ± 0.2^c^

Values with different letters are significantly different from each other (ANOVA followed by Tukey’s post hoc multiple range test, *p* < 0.05). The triple best fits were selected by CDOCER analysis using discovery studio 4.0. SFN, a representative antioxidant, and a C151 residue modifier, was used as a positive control. The significance was analyzed by the post hoc Turkey method (* *p* < 0.05, ** *p* < 0.01, *** *p* < 0.001 compared to the wild type of Keap1 in CDOCKER energy, ^#^ *p* < 0.05, ^##^ *p* < 0.01, ^###^ *p* < 0.001 compared to the wild type of Keap1 in CDOCKER interaction energy)

## Discussion

The phytochemical study suggests that the aerial part of *G. laxum* is an abundant source of natural phenolics, which were identified as quercetin (**2**), quercetin analogues (**3**–**9**), benzoic acid (**10**) and benzoic acid analogues (**1**, **11**). It is well known that phenolics have a wide impact on the living system and that the most interested property of phenolics is antioxidant property [27].

In this study, we used oxidative glutamate toxicity model for determination of neuroprotective effects by quercetin, benzoic acid and their analogues. Oxidative glutamate toxicity is categorized by oxytosis which is a unique oxidative stress-induced programmed cell death, and also it is accompanied by glutathione depletion due to the inhibition of cystine uptake via the cystine/glutamate antiporter [28]. Compound **2**, **4**, **6** and **11** exerted strong protective effects against the oxidative glutamate toxicity.

The Nrf2/Keap1-mediated HO-1 pathway is a possible mechanism for understanding the protective effects of quercetin, benzoic acid and their analogues. It has been well known that Nrf2 regulates the antioxidant enzymes including HO-1 through binding to ARE. Under the condition of oxidative stress, Nrf2 detaches from Keap1 due to the conformation change of Nrf2-Keap1 complex and then released Nrf2 moves to the nucleus, binds to the promoter region of ARE site [29]. Previously, many studies have reported that quercetin and its analogues are effectively scavenged oxy-radicals responsible for the pathogenesis of many diseases, including cancer, neurodegenerative, and cardiovascular diseases, and ageing [30–32]. The neuroprotective and Nrf2-mediated HO-1-inducing activities by quercetin were reported on HT22 cells [33] and its hippocampus effects on hippocampus-dependent learning and memory in mice fed with different diets related with oxidative stress [34].

Quercetin is a typical flavonol and is abundant in many commonly consumed fruits and vegetables, particularly apples, cranberries, blueberries, and onions. A number of studies suggested that antioxidant properties may be responsible for pharmacological activities of quercetin [22]. This observation in the result of a TOSC assay reveals that substitution of methoxy moiety in B ring may be responsible for the decrease of antioxidant activity against peroxyl radicals. Moreover, this is interesting that the presence of a hydroxy group at the 3′ position of B ring is more important than the 4′ position for antioxidant activities.

The cysteine residues in the BTB domain of Keap1, which play the key role in dimerization of Keap1, are covalently modified by oxidative or electrophilic molecules. The modification of disulfide bonds in cysteine residues of the BTB domain in Keap1 leads to dissociation of Cul3, and additionally releases Nrf2. Moreover, the stability of Nrf2 gets robust by decreasing ubiquitination-mediated protein degradation in proteasome [16]. The previous report suggested that C151 is essential for the biological action of sulforaphane (SFN), a representative antioxidant enriched in broccoli, as well as SFN reacts with four cysteine residues in Keap1 including C151 and thereby it modifies the C151 residue in Keap1 [35]. The results of in silico 3D molecular docking simulation indicate that the hydroxy and methoxy groups are important for Keap1 stability by influencing on docking to BTB domain. Compound **3** was stably fit than compound **4** which means that a hydroxy group in B ring also has critical role in docking to BTB domain. On the contrast, the CDOCKER interaction energy had shown in compound **8** < **4** < **3** < **2** and **5** < **7** < **6** < **9** in ascending power. When it compared to compounds **3** and **6**, docking energy of compound **3** was much lower than compound **6** while interaction energy of compound **3** was similar to compound **6**. Overall, hydroxy group in quercetin and its analogues involves in the Keap1 stability by improving to the docking of compounds in BTB domain, and methoxy group enhances the interaction of compounds with BTB domain of Keap1 (Table 2).

## Conclusions

Taken together, the investigation of the structure-active relationship between quercetin and its analogues and antioxidant and neuroprotective activities related to Keap1 showed that the essential role of hydroxy and methoxy groups in B ring of flavonol-type compounds. This study investigated for the first time the significant antioxidant and neuroprotective properties as well as the principles of the aerial part of *G. laxum*. The finding demonstrates that the *G. laxum* can be considered as an abundant natural antioxidant source so that it could be used beneficially in reducing oxidative stress complications.

## Additional files

**Additional file 1.** Minimum Standards of Reporting Checklist.

**Additional file 2.** Additional data.

## Abbreviations

*G. laxum*: *Gynostemma laxum*
ROS: reactive oxygen species
ARE: antioxidant response element
Nrf2: nuclear factor (erythroid-derived 2)-like 2
HO-1: heme oxygenase-1
BTB: N-terminal broad complex, tramtrack and bric-a-brac
Keap1: Kelch-like ECH-associated protein 1
Cul3: cullin 3
TOSC: total oxidant scavenging capacity
ABAP: 2,2′-azobis-amidinopropane
KMBA: α-keto-γ-methiobutyric acid
FID: flame ionizing detector
PDB: protein data bank
DCFDA: 2,7-dichlorofluorescein
DHE: dihydroethidium
