# Supplementary material for: Chemical constituents from a Gynostemma laxum and their antioxidant and neuroprotective activities
Source: Chin Med. 2017 May 24;12:15. doi: 10.1186/s13020-017-0136-y (PMC5442659; doi:10.1186/s13020-017-0136-y)
Supplement: Supplementary file 1 — Additional file 1. Minimum Standards of Reporting Checklist. [file 13020_2017_136_MOESM1_ESM.docx]

Minimum Standards of Reporting Checklist

*BioMed Central* advocates full and transparent reporting. Please ensure that your paper provides the information requested below where applicable. On submitting your paper you will be asked to confirm you have included this information, or give reasons for any instances where it is not made available. You will also be asked to upload this file and it should be cited in the Methods section.

# Experimental design and statistics

The following information should be included in the Methods section and inserted in the table below:

| **Question** | **Answer** |
| --- | --- |
| 1. The exact sample size (n) for each experimental group/condition (as a number, not a range). Include details of a power analysis if done, or any other relevant considerations that determined the choice of sample size. For n < 6, individual data values should be shown rather than summary statistics alone. | We mentioned "Most of data are presented as the means ± SD of duplicates or triplicates (n=2-3)." in statistical analysis part of methods section. To enhance the readers understanding, the exact sample size (n) was written in figure legend section individually.  The Western blot analysis results were shown in the figures. In the case of immunofluorescence and immunocytochemistry results, all figures were in supplementary data because of the figure size.  All repeated results were shown in supplementary data. |
| 2. A description of sample collection that enables the reader to understand whether the samples represent technical or biological replicates, and an explanation of inclusion/exclusion criteria if samples or organisms were excluded from the analysis. | *Gynnostemma laxum* was collected at March. 2013 in Vietnam. Compounds **1**-**11** were provided by Korea Bioactive Natural Material Bank. For the exact plant authentication, we firstly identified with morphological characteristics. We did DNA authentication by comparing the sequence of ITS region of our plant and Genebank NCBI (KF269126) sequence.  Cell lines were used at most of experiments in this study and the descriptions of them were mentioned in ‘Methods’ section of this manuscript. |
| 3. How samples/ organisms were allocated to experimental groups and processed, and full details of the randomisation procedure used (if relevant). | We divided sample groups by concentrations of treatment compounds. (Vehicle, quercetin, quercetin analogues, benzoic acid, benzoic acid analogues by concentrations).  For the cell experiments, the cell density was carefully explained to in the manuscript. |
| 4. For sample assessment by human investigators, a statement on whether the investigator was blinded to group assignment and outcome assessment, and how this blinding was achieved and evaluated (if relevant). | Not applicable |

| 5. How many times each experiment shown was replicated and an indication of the extent of variation from experiment to experiment. | Most of the experiments were done duplicates or triplicates. |
| --- | --- |
| 6. Information on the statistical methods and measures used. It should be clear whether the tests are one-sided or two-sided, whether there are adjustments for multiple comparisons, whether medians or means are being shown, whether error bars are standard deviations (SD), standard error of mean (SEM) or confidence intervals. | We mentioned the answer for this question in the 'methods' section as same as below.  Statistical calculations were examined by one-way analysis of variance (ANOVA), followed by Tukey’s range test, conducting in SPSS Statistics 23 (SPSS, Inc., Chicago, IL, USA).  The data are presented as the means ± standard error (SE) of triplicates or quadruplicates (n=3-4). |
| 7. A justification for the appropriateness of statistical tests used to assess significance. Do the data meet the assumptions of the tests? Is there an estimate of variation within each group of data, and is the variance similar between groups that are being statistically compared?  In addition, information essential to interpreting the data presented should be made available in the figure and table legends. If the study involves health interventions for human participants, please refer to the relevant reporting guidelines from the EQUATOR Network, and the Biosharing Portal for reporting checklists for biological and biomedical research, where applicable. | Yes. The values were compared statistically based on the medians variance analysis.  The essential interpretation for the data was implied in the titles of figures.  We did not prepare our data as the tools of clinical studies with human participants. Our results were come the biological activities with compounds **1**-**11** from *Gynostemma laxum*.  These results suggest that this plant was used for reducing oxidative complications with the further investigations as the plentiful contents of compounds **1**-**11**. |

# Research involving humans

If your research involved humans, please confirm you have adhered to the relevant reporting guideline from the [EQUATOR Network](http://www.equator-network.org/), and included the completed checklist as an additional file with your submission:

|  | **Answer** (page and line number inserted/Not applicable for my study) |
| --- | --- |
| - I have followed the relevant reporting for my study type, and included a populated checklist with my submission - Not applicable for my study | Not applicable for my study.  We did not use any human relevant reporting in this manuscript. |

# Resources

A description of all resources used should be included in the Methods section, with enough information to allow them to be uniquely identified. The table below should be completed with confirmation that this was done (i.e. included in the Methods section) or is not applicable. If this has not been completed, but is applicable, you should contact the journal editorial staff before proceeding.

|  | **Answer** (page and line number inserted/Not applicable for my study) |
| --- | --- |
| •Antibodies: report source, catalogue code, characteristics, dilutions and how they were validated for the system under study. | page 10, line number 173-174  After blocking the non-specific proteins on the membrane, the primary antibodies for Nrf2 (H-300, Santa Cruz), HO-1 (sc-1797, Santa Cruz), Lamin B (sc-474, Santa Cruz) and β-actin (ab6276, Abcam) were loaded and were incubated for more than 18 h at 4°C. |
| •Cell lines: report source, whether identity has been authenticated and whether tested for mycoplasma contamination. We encourage researchers to check the NCBI database for contamination of cell lines. | page 9, line number 163  HT22-ARE or SH-SY5Y-ARE cells [[25](#_ENREF_24)] were plated onto 12-well plates at a density of 3 × 10^5^ cells/well for 6 h, and then the cells were incubated in DMEM containing 1% FBS for another 6 h.  All used cell lines were tested for mycoplasma contamination. Our cell lines were not contaminated by any mycoplasma. |
| •Organisms: report source, species, strain, sex, age, husbandry, inbred and strain characteristics of transgenic and mutant animals. | It is not applicable for my study.  We did not use any animals in this study. |
| •Tools (software, databases and services): report standard tool name, provider and version number, if available. For antibodies, model organisms (mice, zebrafish and flies) and tools, authors are strongly encouraged to cite Research Resource Identifiers (RRIDs). To do so, please go to the Resource Identification Portal to search for your research resource and insert the reference text into your Methods section. | Page 9, line number 155.  **Antioxidant response element (ARE)-reporter gene assay.** The luciferase activity was measured according to the protocol guided by the manufacturer (Promega Corp., WI).  Page 10, line number 181.  **Real-time PCR.** The mRNA extraction was performed by the Trizol method and then the mRNA was immediately synthesized to cDNA using Maxim RT PreMix (random primer) manufactured from iNtRON Biotechnology (Seongnam, Korea).  Page 12, line number 204-205.  **Chemical structures** of isolated compounds were identified by ^1^H, ^13^C, and HMBC NMR analyses, and comparing their physicochemical and spectroscopic data with those published in literatures (Supplementary data). |

# Availability of data and materials

The table below should be completed with confirmation that this was done (i.e. included in the Methods section) or is not applicable.

|  | **Answer** (page and line number inserted/Not applicable for my study) |
| --- | --- |
| All datasets on which the conclusions of the paper rely must be either deposited in publicly available repositories (where available and ethically appropriate) or presented in the main paper or additional supporting files, in machine-readable format whenever possible. If authors are unable to fulfil this requirement, they should contact journal editorial staff, after checking our list of Recommended Repositories. | We attached the supporting file (supplementary data). |
| Links to deposited datasets, or datasets in additional files, should be explicitly referenced in a section entitled “Availability of Data and Materials”. Guidance on where to deposit your data can be found on the Availability of Data and Materials policy page. | It is not applicable for my study. |
| If computer code was used to generate results that are central to the paper’s conclusions, include a statement in the “Availability of data and materials” section to indicate how the code can be accessed. Include version information and any restrictions on availability. For deposited data and published code, a full reference with an accession number, doi or other unique identifier should be included in the reference list. | It is not applicable for my study. |
| If reproducible materials are generated as a result of the research (for example new animal mutants), a statement on their availability should be included. | Compounds **1**-**11** can be isolated and characterized from *G. laxum* by researcher himself. After he isolated these compounds **1**-**11**, he can confirm his compounds by comparing of physico-chemical properties of the supplementary data |
