# Supplementary material for: Chemical constituents from a Gynostemma laxum and their antioxidant and neuroprotective activities
Source: Chin Med. 2017 May 24;12:15. doi: 10.1186/s13020-017-0136-y (PMC5442659; doi:10.1186/s13020-017-0136-y)
Supplement: Supplementary file 2 — Additional file 2. Additional data. [file 13020_2017_136_MOESM2_ESM.doc]

**Additional data 2**

**Chemical constituents from a *Gynostemma laxum* and their antioxidant and neuroprotective activities**

Ji Yeon Seo^1^, Sang Kyum Kim^2^, Phi Hung Nguyen^3^, Ju Yong Lee^1^, Pham Ha Thanh Tung^1^, Sang Hyun Sung^1^, and Won Keun Oh^*,1^

^1^Korea Bioactive Natural Material Bank, Research Institute of Pharmaceutical Sciences, College of Pharmacy, Seoul National University, Seoul 08826, Republic of Korea; [quftkfka@gmail.com](mailto:quftkfka@gmail.com) (J.Y.S.); [sbplee@snu.ac.kr](mailto:sbplee@snu.ac.kr) (J.Y.L.); [thtungdl@snu.ac.kr](mailto:thtungdl@snu.ac.kr) (P.H.T.T.); [shsung@snu.ac.kr](mailto:shsung@snu.ac.kr) (S.H.S.); [wkoh1@snu.ac.kr](mailto:wkoh1@snu.ac.kr) (W.K.O.).

^2^College of Pharmacy, Chungnam National University, Daejeon 34134, Republic of Korea; [sangkim@cnu.ac.kr](mailto:sangkim@cnu.ac.kr) (S.K.K.).

^3^College of Pharmacy, Chosun University, Gwangju 61452, Republic of Korea; [hungcbt1980@yahoo.com](mailto:hungcbt1980@yahoo.com) (P.H.N.).

A short title : Antioxidant and neuroprotective effects of quercetin analogues

^*^ To whom correspondence should be addressed. Tel & Fax: +82-02-880-7872. E-mail: [wkoh1@snu.ac.kr](mailto:wkoh1@snu.ac.kr).

**Contents:**

**Table**

**Table S1.** ^1^H (500 MHz) NMR data of isolated quercetin-type compounds **2−9** from *Gynostemma laxum* in acetone-*d_6_*

**Figures**

**Figure S1.** Morphological and DNA authentication of *G. laxum*.

**Figure S2.** Physico-chemical properties of phenolic compounds **1**-**11**from *G. laxum*.

**Figure S3.** A representative HPLC profile of total phenolic compounds **1**−**11** from the EtOAc fraction of the 70%-EtOH extract of *G. laxum.* Key to peak identity: **1** (3,4-dihydroxybenzoic acid, *t*_R_ 12.1 min), **2** (quercetin, *t*_R_ 25.8 min), **3** (quercetin-3'-methyl ether, *t*_R_ 30.0 min), **4** (quercetin-4'-methyl ether, *t*_R_ 36.1 min), **5** (quercetin-3,4'-dimethyl ether, *t*_R_ 44.9 min), **6** (quercetin-3,3'-dimethyl ether, *t*_R_ 48.7 min), **7** (ermanin, *t*_R_ 58.2 min), **8** (quercetin-3',4'-dimethyl ether, *t*_R_ 28.2 min), **9** (kaempferol-3-methyl ether, *t*_R_ 47.7 min), **10** (benzoic acid, *t*_R_ 23.1 min), and **11** (3-ethoxy-4-hydroxybenzoic acid, *t*_R_ 19.8 min).

**Figure S4**. Protective effects of various concentrations of quercetin on cell death induced by the excessive amount of glutamate in HT22 cells. Cells were seeded at a density of 5 × 10^3^ cells per each well onto a 96-well plate. After 2 h, the cells were co-treated with 0, 5, or 10 mM of glutamate and quercetin in a range of concentrations 1.25, 2.5, 5, 10, 40, and 80 μM in DMEM containing 5% FBS and P/S for 12-14 h depending on the cell death. The cell viability was assessed by a MTT reduction assay.

**Figure S5**. Induction of ARE transcriptional activity by isolated compounds **1**−**11**. **(A)** HT22-ARE cells. **(B)** SHSY5Y-ARE cells.

**Figure S6**. The protein expressions of nuclear Nrf2 or HO-1 regulated by compound **4** in HT22 cells. The expression levels of nuclear Nrf2, nuclear Lamin B, HO-1 and β-actin were analysed by Western blotting in duplicates.

**Figure S7**. Effect of compound **4** on nuclear translocation of Nrf2 and the expression of Keap1 in HT22 cells. The HT22 cells stained by anti-Nrf2, DAPI, and anti-Keap1 and visualized by confocal fluorescence microscope.

**Figure S8**. *In silico* molecular docking simulation of isolated compounds **1**-**11** against BTB domain of Keap1. **(A)** 3D molecular docking simulation results of benzoic acid (**10**) and its analogues (**1** and **11**). **(B)** 2D diagram results about non-covalent bonding interactions of benzoic acid (**10**) and its analogues (**1** and **11**). **(C)** 3D molecular docking simulation results of quercetin (**2**) and its analogues (**3**-**9**). **(D)** 2D diagram results about non-covalent bonding interactions of quercetin (**2**) and its analogues (**3**-**9**).

**Figure S9**. *In silico* molecular docking simulation of isolated compounds **1**-**11** against C151W mutant at BTB domain of Keap1. **(A)** 3D molecular docking simulation results of quercetin (**2**) and its analogues (**3**-**9**). **(B)** 2D diagram results about non-covalent bonding interactions of quercetin (**2**) and its analogues (**3**-**9**).

**References**

***Table S1.*** *1H (500 MHz) NMR data of isolated quercetin-type compounds* ***2−9*** *from Gynostemma laxum in acetone-d*6

| **No.** | **2** |  | **3** |  | **4** |  | **5** |  | **6** |  | **7** |  | **9** |
| --- | --- | --- | --- | --- | --- | --- | --- | --- | --- | --- | --- | --- | --- |
|  | δ_H_ (*J* in Hz) |  | δ_H_ (*J* in Hz) |  | δ_H_ (*J* in Hz) |  | δ_H_ (*J* in Hz) |  | δ_H_ (*J* in Hz) |  | δ_H_ (*J* in Hz) |  | δ_H_ (*J* in Hz) |
| 1 |  |  |  |  |  |  |  |  |  |  |  |  |  |
| 2 |  |  |  |  |  |  |  |  |  |  |  |  |  |
| 3 |  |  |  |  |  |  |  |  |  |  |  |  |  |
| 4 |  |  |  |  |  |  |  |  |  |  |  |  |  |
| 5 |  |  |  |  |  |  |  |  |  |  |  |  |  |
| 6 | 6.25, d, 2.0 |  | 6.27, d, 1.5 |  | 6.31, d, 1.5 |  | 6.31, d, 2.0 |  | 6.31, d, 2.0 |  | 6.32, d, 2.0 |  | 6.20, d, 2.0 |
| 7 |  |  |  |  |  |  |  |  |  |  |  |  |  |
| 8 | 6.50, d, 2.0 |  | 6.55, d, 1.5 |  | 6.68, d, 1.5 |  | 6.72, d, 2.0 |  | 6.70, d, 2.0 |  | 6.71, d, 2.0 |  | 6.58, d, 2.0 |
| 9 |  |  |  |  |  |  |  |  |  |  |  |  |  |
| 10 |  |  |  |  |  |  |  |  |  |  |  |  |  |
| 1′ |  |  |  |  |  |  |  |  |  |  |  |  |  |
| 2′ | 7.81, d, 2.0 |  | 7.88, d, 2.5 |  | 7.83, d, 1.5 |  | 7.84, d, 1.5 |  | 7.90, d, 2.5 |  | 8.25, br, d, 9.0 |  | 8.05, br, d, 9.0 |
| 3′ |  |  |  |  |  |  |  |  |  |  | 7.12, br, d, 9.5 |  | 6.90, br, d, 9.0 |
| 4′ |  |  |  |  |  |  |  |  |  |  |  |  |  |
| 5′ | 6.98, d, 8.5 |  | 7.00, d, 9.0 |  | 7.00, d, 8.5 |  | 7.12, d, 8.0 |  | 7.00, d, 8.5 |  | 7.12, br, d, 9.5 |  | 6.90, br, d, 9.0 |
| 6′ | 7.68, dd, 2.0, 8.5 |  | 7.81, dd, 2.5, 8.5 |  | 7.71, dd, 1.5, 8.5 |  | 7.81, dd, 1.5, 8.0 |  | 7.83, dd, 2.5, 8.5 |  | 8.25, br, d, 9.0 |  | 8.05, br, d, 9.0 |
| 3-OMe |  |  |  |  |  |  | 3.90, s |  | 3.89, s |  | 3.89, s |  | 3.83, s |
| 3′-OMe |  |  |  |  | 3.93, s |  |  |  | 3.92, s |  |  |  |  |
| 4′-OMe |  |  | 3.93, s |  |  |  | 3.93, s |  |  |  | 3.92, s |  |  |
| 5-OH | 12.15, s |  | 12.15, s |  | 12.13, s |  | 12.09, s |  | 12.11, s |  | 12.11, s |  | 12.01, s |

**Figure S1**. Morphological and DNA authentication of *G. Laxum*. (**A**) Authentication of *G. laxum* based on morphology and nuclear ribosomal internal transcribed spacer (ITS) region. (1) Whole plant with abaxial leaves, (2) abaxial leaf, (3) male flower, (4) female flower, (5) infructescence, (6) seed. B. Genetic information for authentication of *G. laxum*.

**
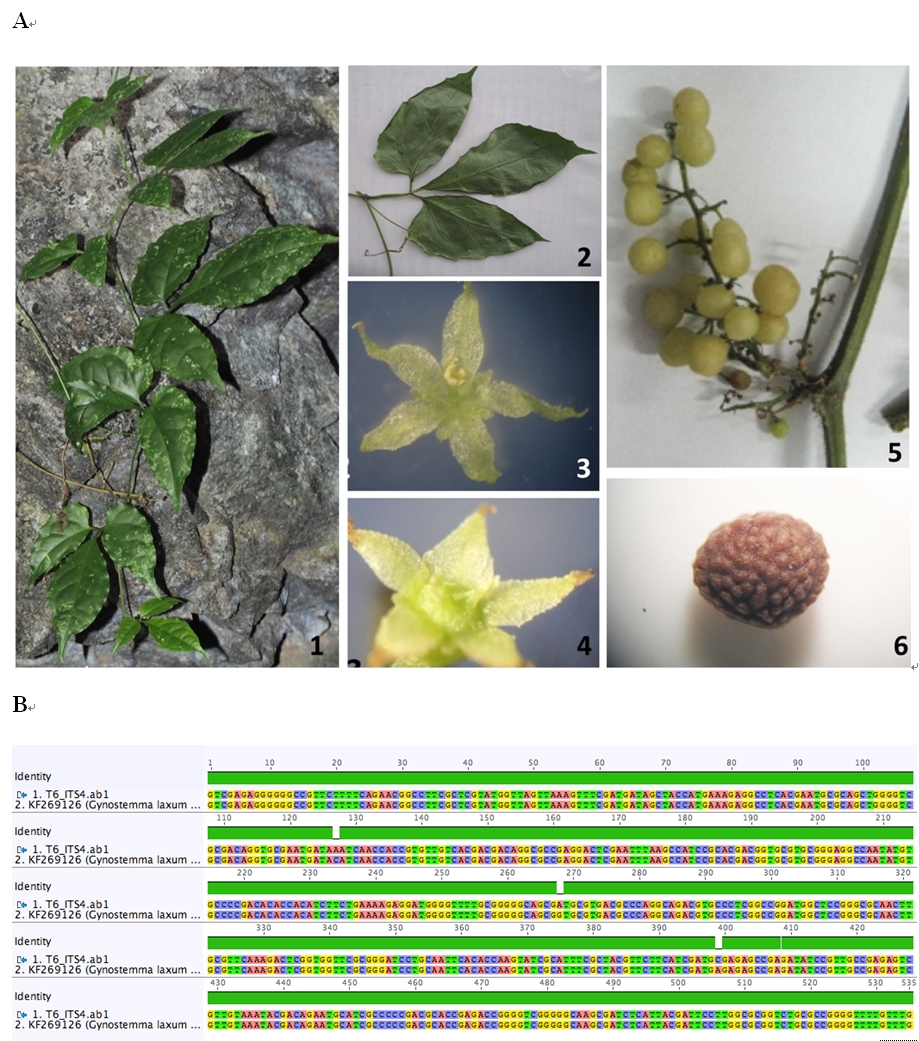
**

**Figure S2.** Physico-chemical properties of phenolic compounds **1**-**11**from *G. laxum*

Chemical structures of isolated compounds were identified by ^1^H, ^13^C, and HMBC NMR analyses, and comparing their physicochemical and spectroscopic data with those published in literatures (Table S1). In summary, these compounds were determined as 3,4-dihydroxybenzoic acid (**1**), quercetin (**2**), quercetin-3'-methyl ether (**3**), quercetin-4'-methyl ether (**4**), quercetin-3,4'-dimethyl ether (**5**), quercetin-3,3'-dimethyl ether (**6**), ermanin (**7**), quercetin-3',4'-dimethyl ether (**8**), kaempferol-3-methyl ether (**9**), benzoic acid (**10**), and 3-ethoxy-4-hydroxybenzoic acid (**11)**.

Quercetin (**2**): Yellow powder; m.p. (uncorrected) 312-318^o^C, FeCl_3_ test: positive; UV (MeOH) λ_max_ nm: 260, 381; ^13^C NMR (125 MHz, acetone-*d*_6_): δ_C_ 178.2, 165.3, 164.1, 158.9, 153.3, 148.5, 147.7, 132.8, 123.9, 120.2, 117.3, 112.1, 104.6, 95.8, 94.9; ^1^H NMR data are in Table S1.

Quercetin-3'-methyl ether (**3**): Yellowish powder; FeCl_3_ test: positive; ^13^C NMR (125 MHz, acetone-*d*_6_): δ_C_ 177.6, 166.7, 162.4, 157.9, 151.2, 148.7, 147.4, 131.8, 124.1, 121.3, 116.8, 113.7, 106.8, 95.9, 95.6, 56.5; ^1^H NMR data are in Table S1.

Quercetin-4'-methyl ether (**4**): Yellowish powder; FeCl_3_ test: positive; ^13^C NMR (125 MHz, acetone-*d*_6_): δ_C_ 178.1, 164.5, 163.1, 158.8, 154.5, 147.4, 147.0, 132.3, 124.1, 120.7, 117.8, 112.9, 105.6, 96.7, 95.6, 56.4; ^1^H NMR data are in Table S1.

Quercetin-3,4'-dimethyl ether (**5**): Yellowish powder; FeCl_3_ test: positive; ^13^C NMR (125 MHz, acetone-*d*_6_): δ_C_ 181.0, 166.6, 163.0, 158.5, 150.3, 147.4, 147.0, 131.5, 123.7, 121.3, 113.6, 112.5, 105.0, 98.5, 92.8, 56.5, 56.4; ^1^H NMR data are in Table S1.

Quercetin-3,3'-dimethyl ether (**6**): Yellowish powder; FeCl_3_ test: positive; ^13^C NMR (125 MHz, acetone-*d*_6_): δ_C_ 182.1, 165.9, 161.0, 157.8, 151.3, 148.1, 147.6, 131.1, 125.1, 119.7, 115.3, 112.2, 108.5, 99.5, 95.6, 56.9, 56.5; ^1^H NMR data are in Table S1.

Ermanin (**7**): Yellowish powder; FeCl_3_ test: positive; ^13^C NMR (125 MHz, acetone-*d*_6_): δ_C_ 183.5, 164.9, 164.8, 163.7, 155.6, 151.2, 131.3, 129.0, 124.6, 115.4, 106.5, 104.2, 91.6, 60.0, 56.9; ^1^H NMR data are in Table S1.

Quercetin-3',4'-dimethyl ether (**8**): Yellowish powder; FeCl_3_ test: positive; ^1^H NMR data (500 MHz, acetone-*d*_6_): δ_H_ 7.91 (1H, d), 7.84 (1H, dd) , 7.00 (1H, d), 6.67 (1H, d), 6.31 (1H, d), 3.92 (6H, br, s); ^13^C NMR (125 MHz, acetone-*d*_6_): δ_C_ 179.9, 164.1, 159.0, 153.4, 153.3, 152.5,149.5, 132.8, 123.9, 120.3, 111.3, 108.9, 106.3, 97.9, 96.6, 56.5, 56.3.

Kaempferol-3-methyl ether (**9**): Yellowish powder; FeCl_3_ test: positive; ^13^C NMR (125 MHz, acetone-*d*_6_): δ_C_ 183.6, 165.0, 163.9, 160.2, 154.3, 154.1, 132.6, 129.2, 124.4, 115.5, 106.6, 104.4, 92.1, 56.8; ^1^H NMR data are in Table S1.

**Figure S3.** A representative HPLC profile of total phenolic compounds **1**−**11** from the EtOAc fraction of the 70%-EtOH extract of *G. laxum.* Key to peak identity: **1** (3,4-dihydroxybenzoic acid, *t*_R_ 12.1 min), **2** (quercetin, *t*_R_ 25.8 min), **3** (quercetin-3'-methyl ether, *t*_R_ 30.0 min), **4** (quercetin-4'-methyl ether, *t*_R_ 36.1 min), **5** (quercetin-3,4'-dimethyl ether, *t*_R_ 44.9 min), **6** (quercetin-3,3'-dimethyl ether, *t*_R_ 48.7 min), **7** (ermanin, *t*_R_ 58.2 min), **8** (quercetin-3',4'-dimethyl ether, *t*_R_ 28.2 min), **9** (kaempferol-3-methyl ether, *t*_R_ 47.7 min), **10** (benzoic acid, *t*_R_ 23.1 min), and **11** (3-ethoxy-4-hydroxybenzoic acid, *t*_R_ 19.8 min).

**1**

**2**

**3**

**4**

**5**

**6**

**11**

**8**

**7**

**10**

**9**

Phytochemical study suggests that the aerial part of *G. laxum* is an abundant source of natural phenolics, which were identified as quercetin derivatives (**2**–**9**) and benzoic acid derivatives (**1** and **10**–**11**). It is well known that phenolics have a wide impact on the living system and that the most interested property of phenolics is antioxidant property.

**Figure S4**. Protective effects of various concentrations of quercetin on cell death induced by the excessive amount of glutamate in HT22 cells. Cells were seeded at a density of 5 × 10^3^ cells per each well onto a 96-well plate. After 2 h, the cells were co-treated with 0, 5, or 10 mM of glutamate and quercetin in a range of concentrations 1.25, 2.5, 5, 10, 40, and 80 μM in DMEM containing 5% FBS and P/S for 12-14 h depending on the cell death. The cell viability was assessed by a MTT reduction assay.


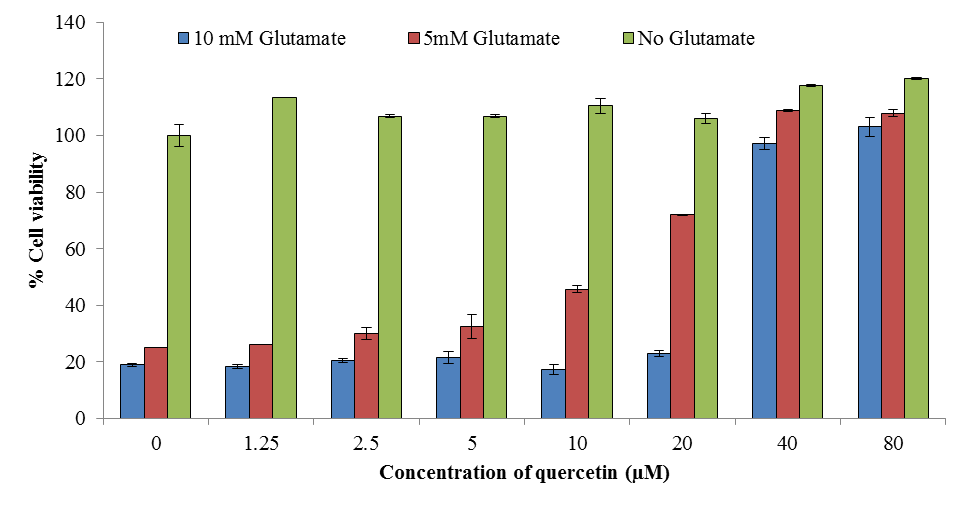


HT22 cells were effectively protected by quercetin treatment against cell death induced by excessive amount (5 or 10 mM) of glutamate treatment. These results indicate that quercetin has the protective effects although the conditions such as cell density and incubation time are changed a little bit. Moreover it has no cytotoxicity at high concentration (40 or 80 μM).

**Figure S5**. Induction of ARE transcriptional activity by isolated compounds **1**−**11**. (**A**) HT22-ARE cells. (**B**) SHSY5Y-ARE cells.

(**A**)
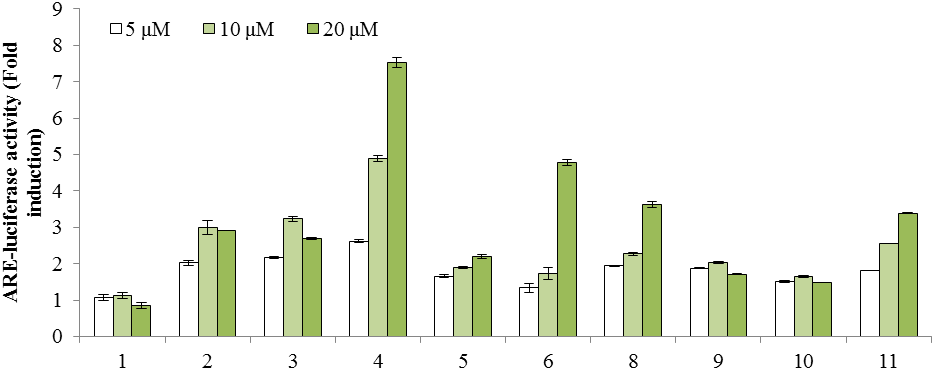


(**B**)
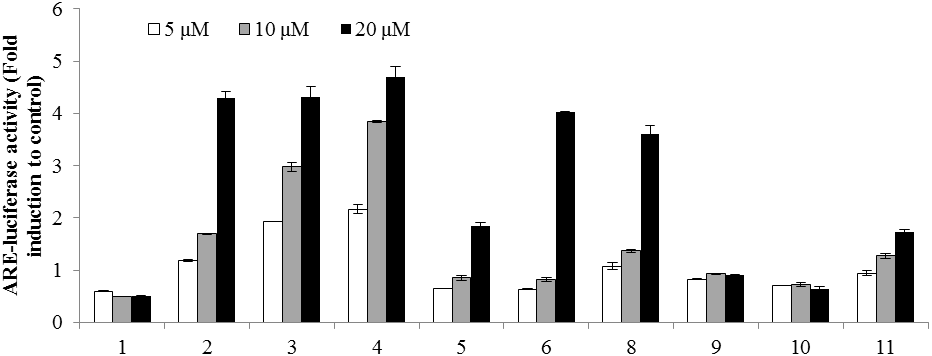


**Figure S6**. The protein expressions of nuclear Nrf2 or HO-1 regulated by compound **4** in HT22 cells. The expression levels of nuclear Nrf2, nuclear Lamin B, HO-1 and β-actin were analysed by Western blotting in duplicates.


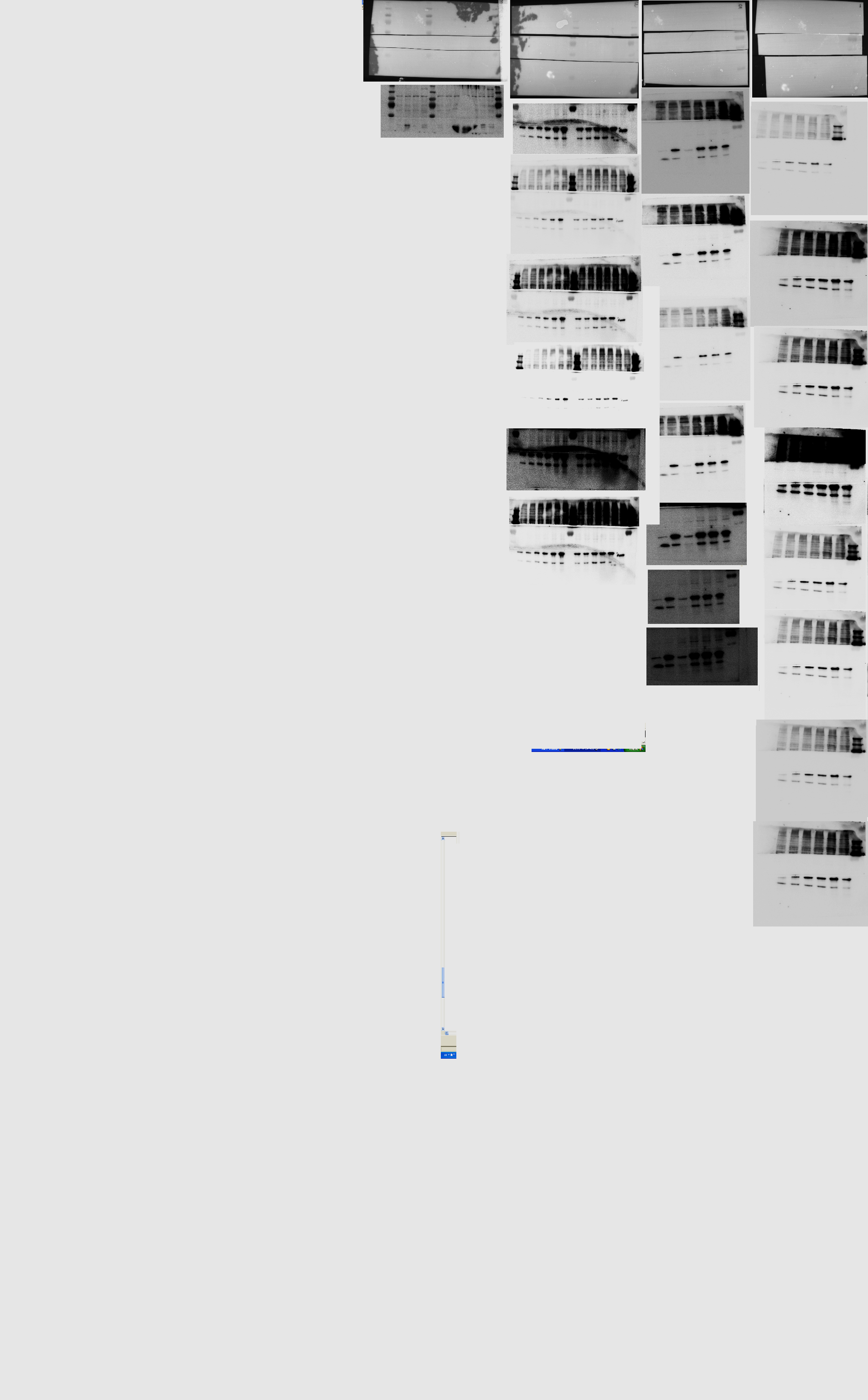

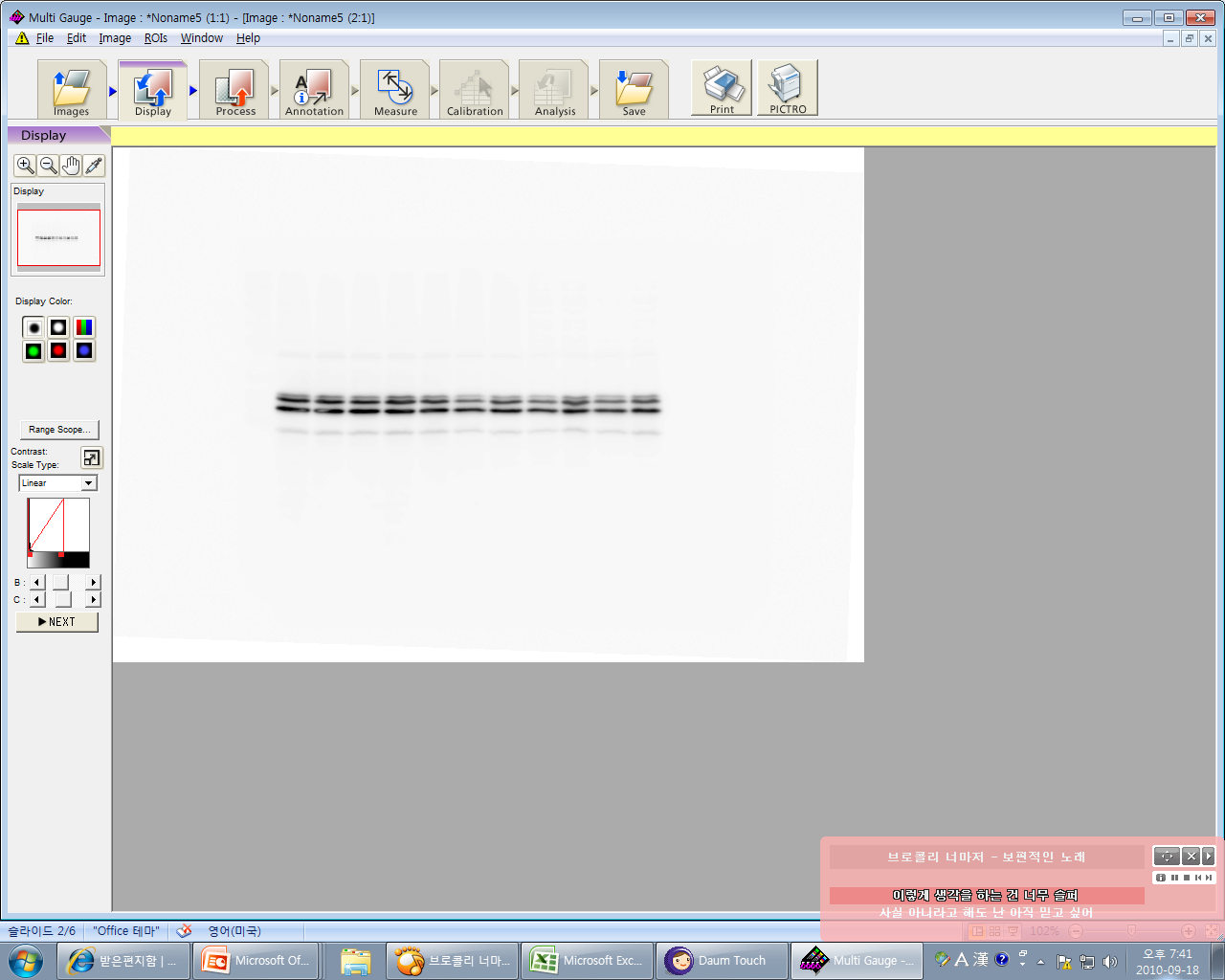

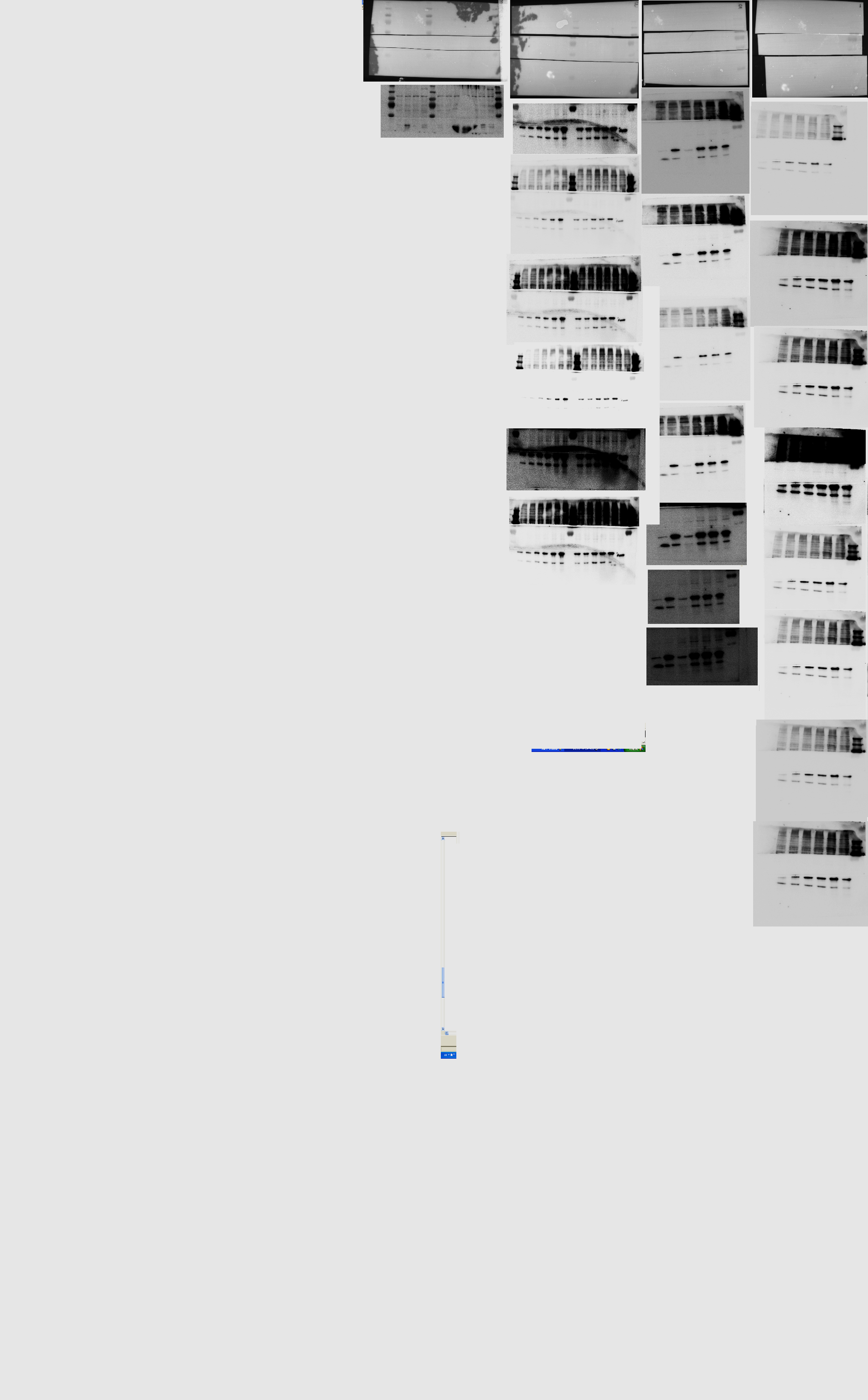

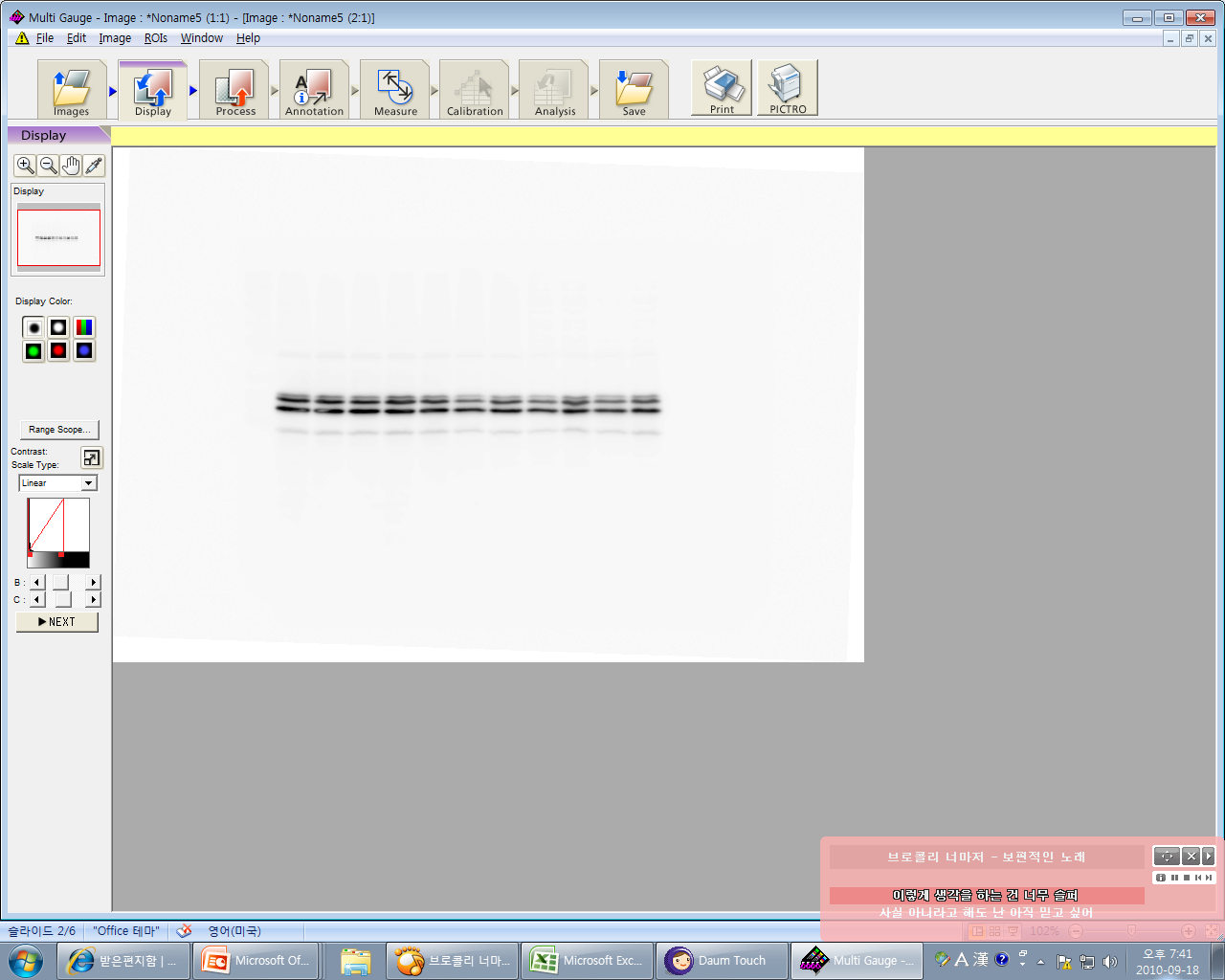


Nuclear Nrf2

Lamin B

-

5

10

20

Q4′ME

-

5

10

20


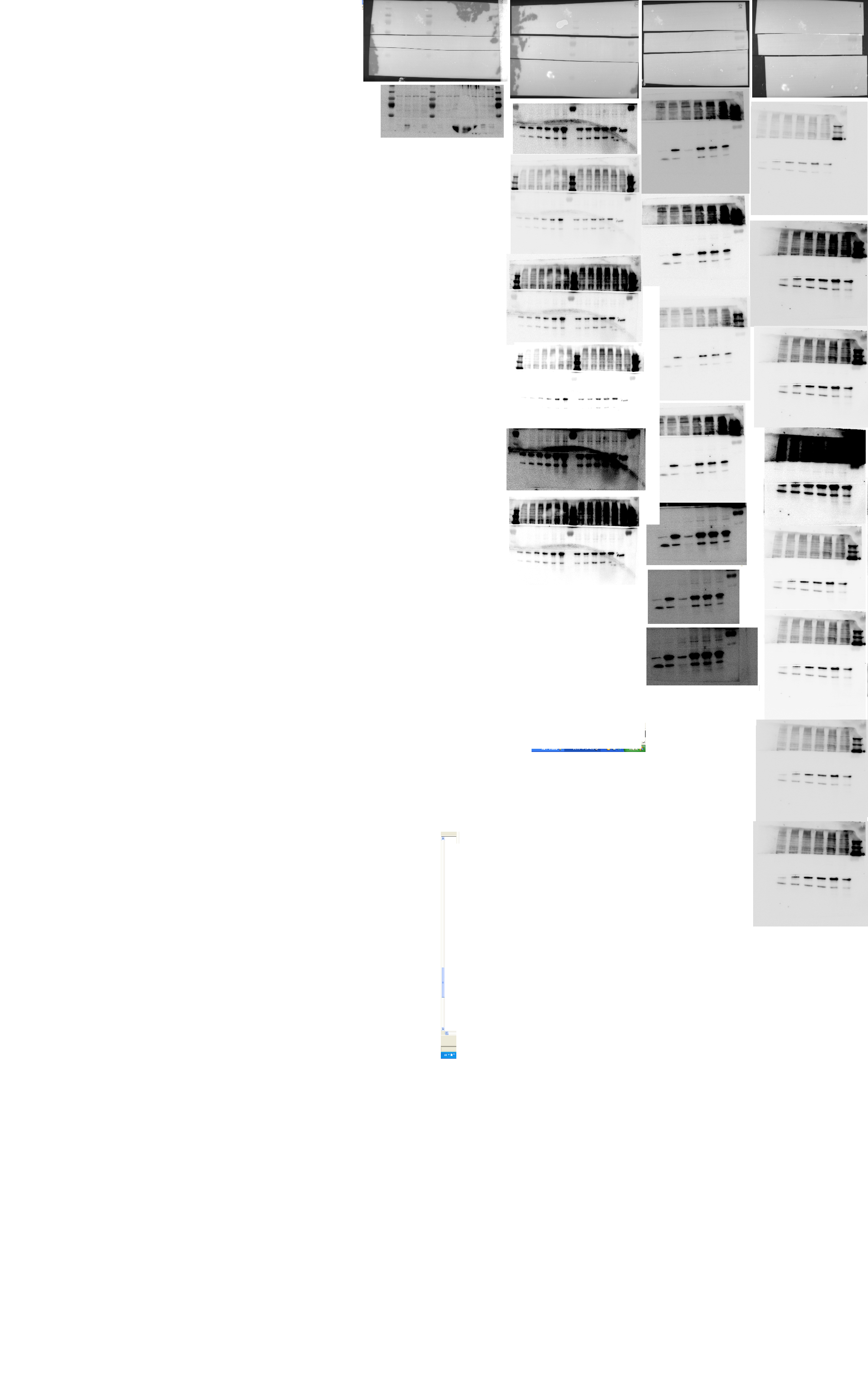

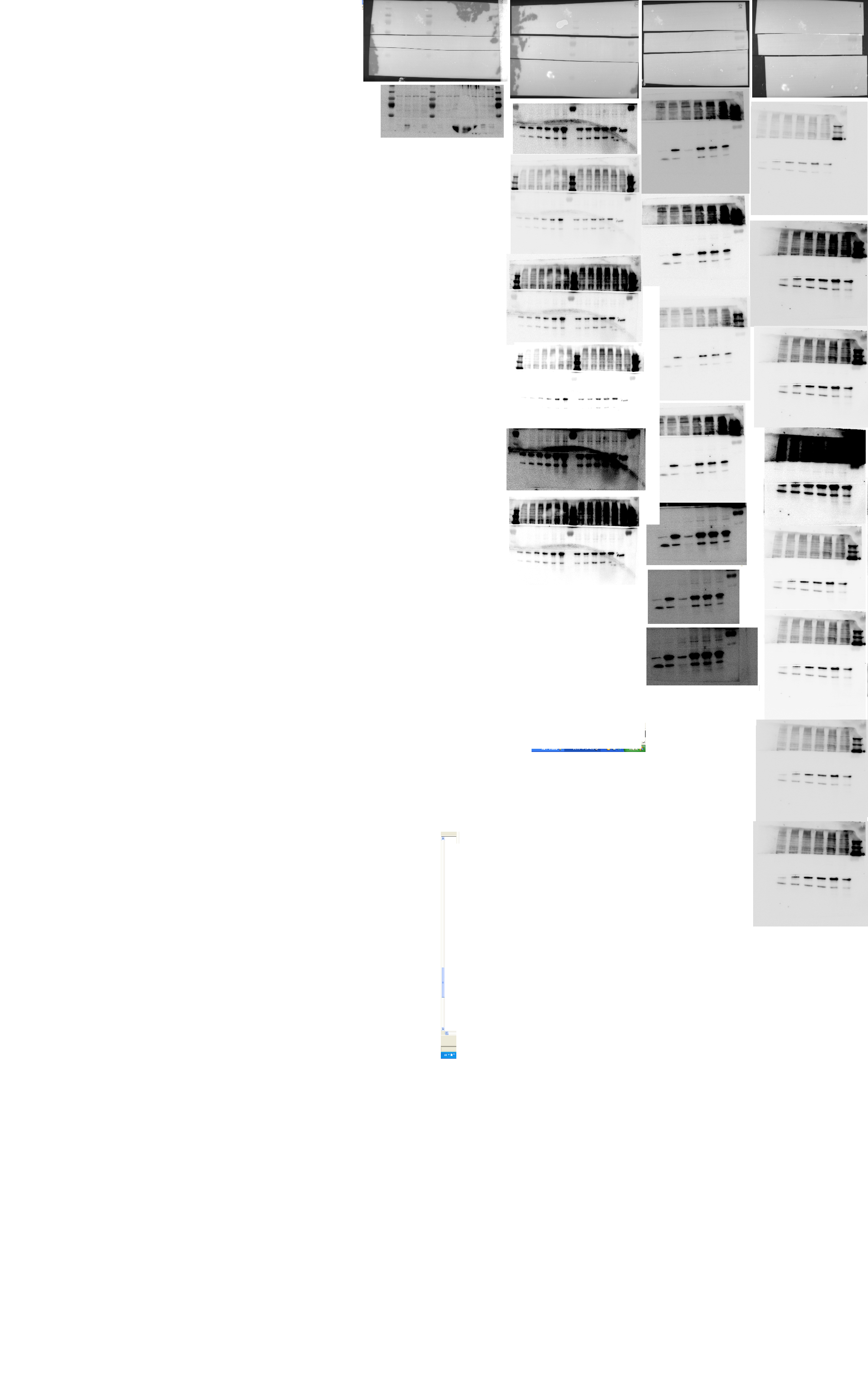

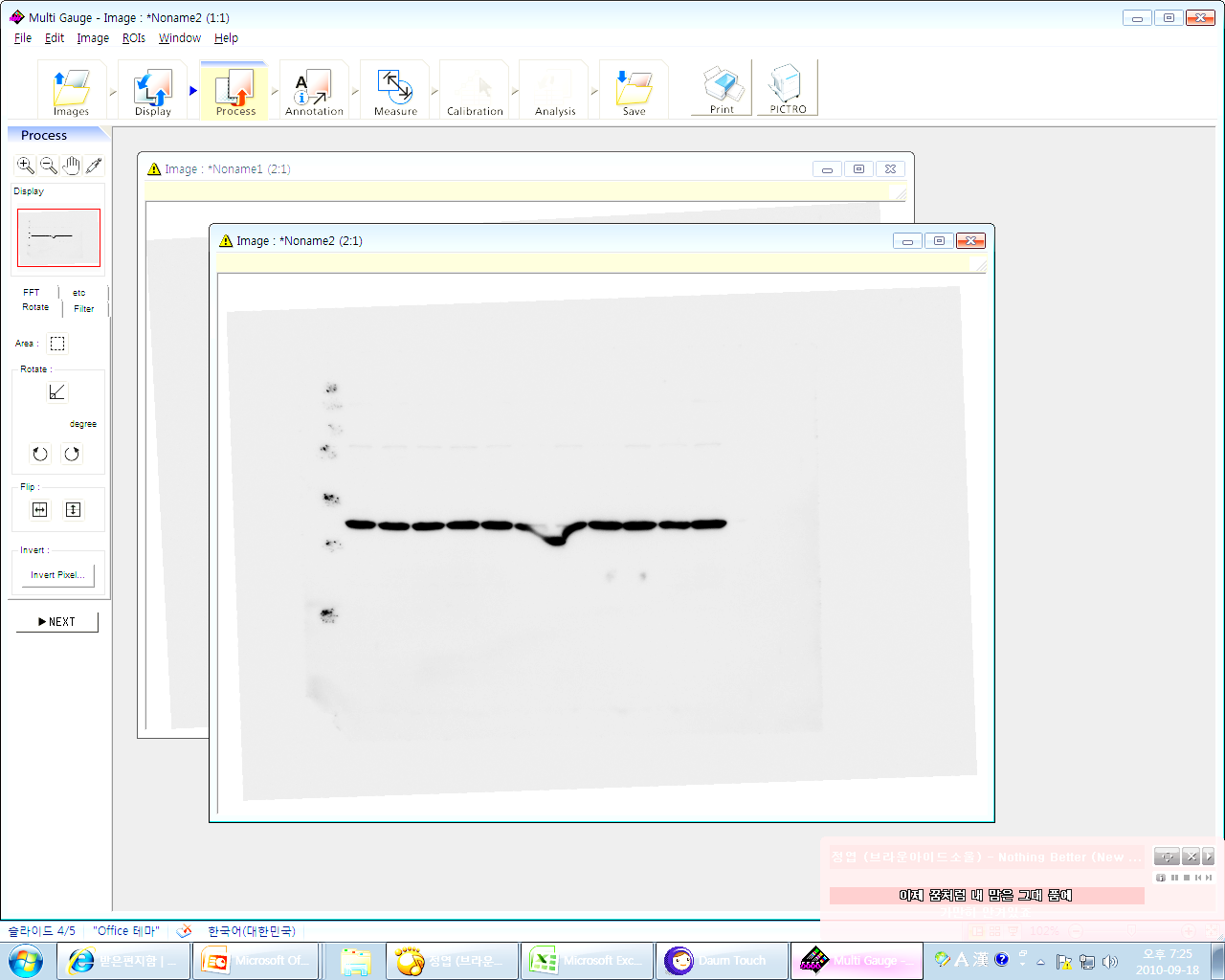

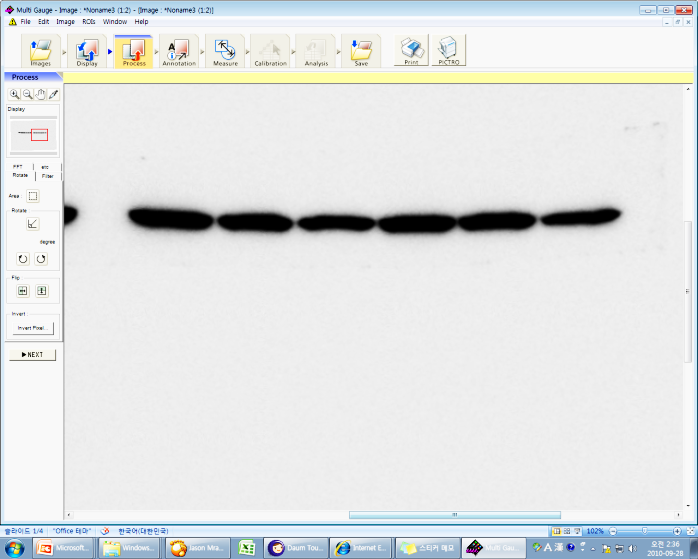


HO-1

β-actin

-

5

10

20

Q4′ME

-

5

10

20

**Figure S7**. Effect of compound **4** on nuclear translocation of Nrf2 and the expression of Keap1 in HT22 cells. The HT22 cells stained by anti-Nrf2, DAPI, and anti-Keap1 and visualized by Confocal fluorescence microscope.


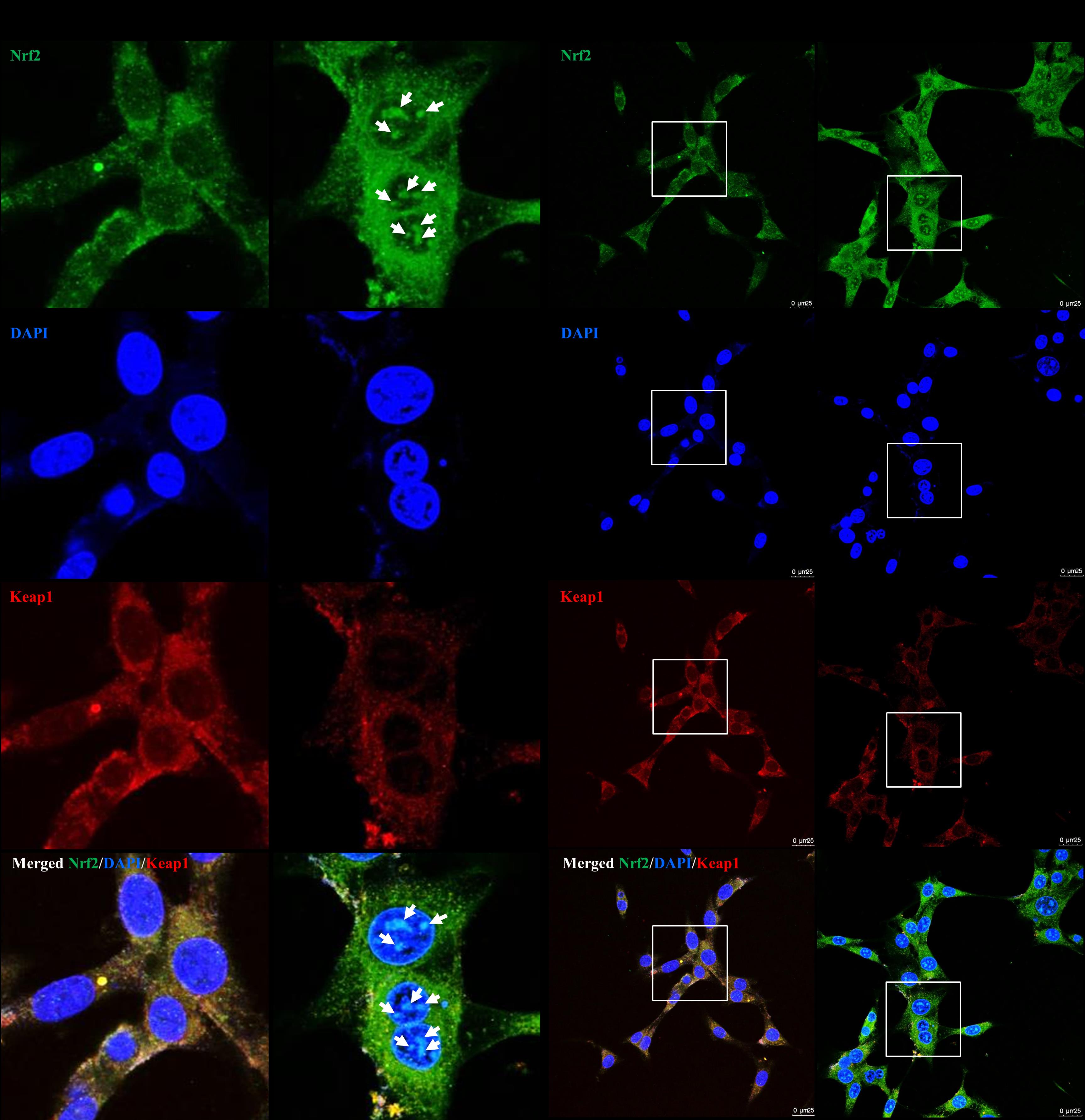


**Figure S8**. *In silico* molecular docking simulation of isolated compounds **1**-**11** against BTB domain of Keap1. (**A**) 3D molecular docking simulation results of benzoic acid (**10**) and its analogues (**1** and **11**). (**B**) 2D diagram results about non-covalent bonding interactions of benzoic acid (**10**) and its analogues (**1** and **11**). (**C**) 3D molecular docking simulation results of quercetin (**2**) and its analogues (**3**-**9**). (**D**) 2D diagram results about non-covalent bonding interactions of quercetin (**2**) and its analogues (**3**-**9**).


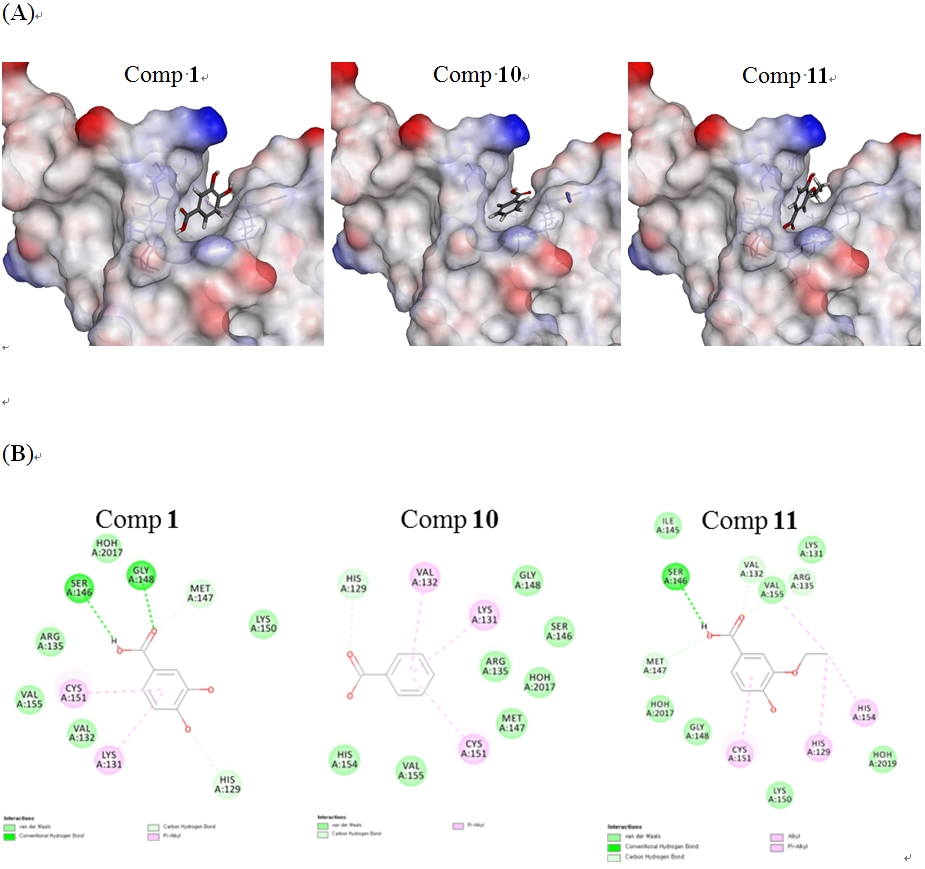


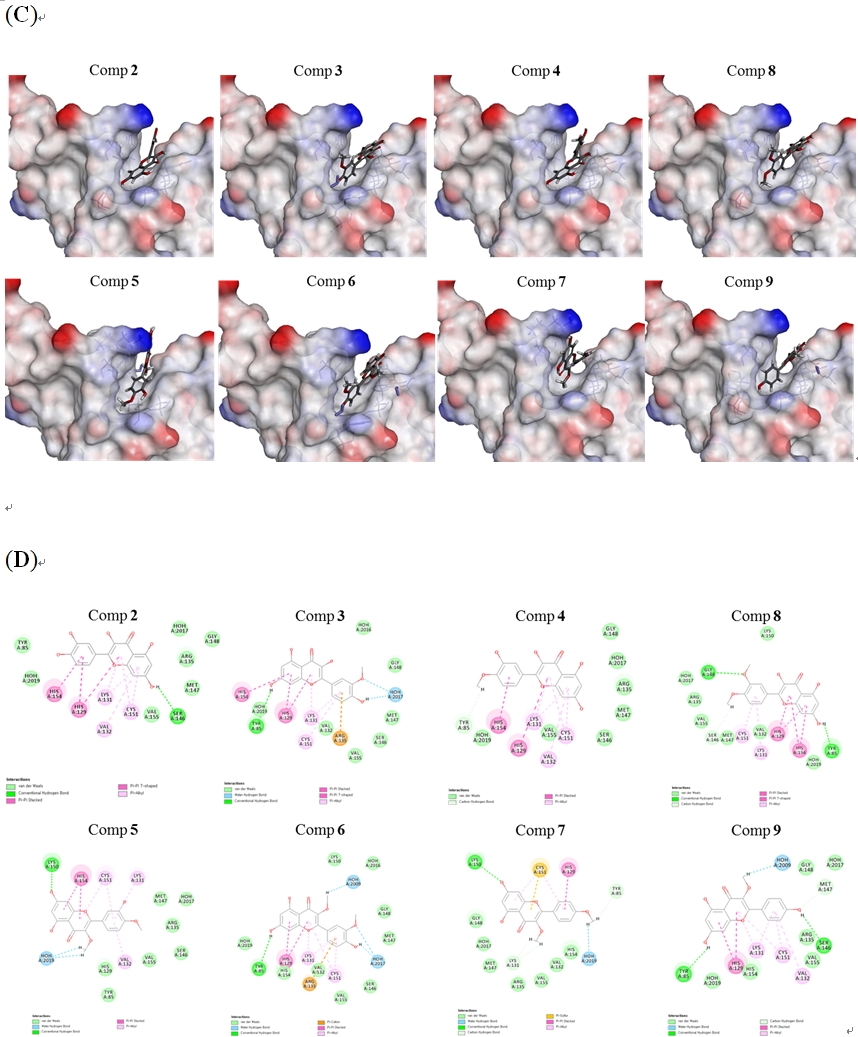


**Figure S9**. *In silico* molecular docking simulation of isolated compounds **1**-**11** against C151W mutant at BTB domain of Keap1. (**A**) 3D molecular docking simulation results of quercetin (**2**) and its analogues (**3**-**9**). (**B**) 2D diagram results about non-covalent bonding interactions of quercetin (**2**) and its analogues (**3**-**9**).


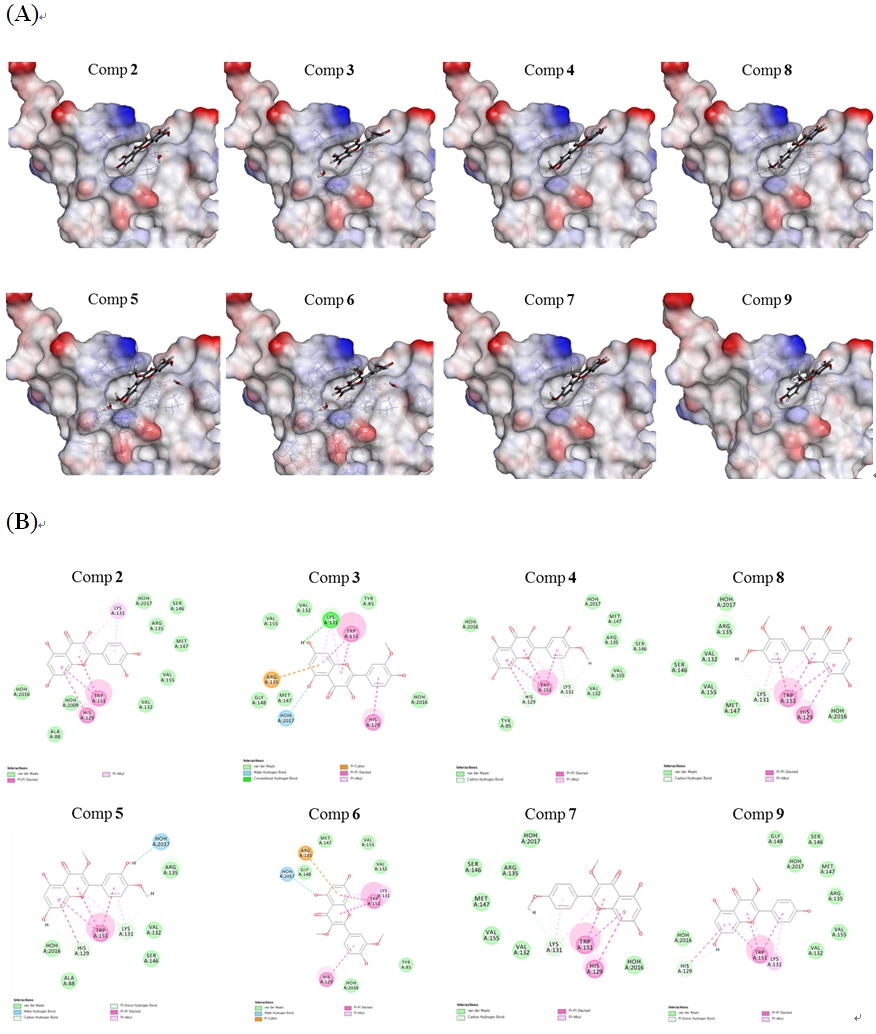


**References**

1. Mean, K. H.; Mohamed, S. Flavonoid (myricetin, quercetin, kaempferol, luteolin, and apigenin) content of edible tropical plants. *J. Agri. Food. Chem.* **2001**, *49*, 3106–3112.

2. Karakaya, S. Bioavailability of phenolic compounds. *Crit. Rev. Food Sci. Nutr.* **2004**, *44*, 453–464.
